# Optimising Guide RNA Production for Multiplexed Cas9-Targeted Nanopore Sequencing to Detect Pathogens

**DOI:** 10.1007/s12033-025-01510-9

**Published:** 2025-09-07

**Authors:** Gus R. McFarlane, Kim Whitaker, Krista L. Plett, Brendon O’Rourke, Daniel R. Bogema

**Affiliations:** NSW Department of Primary Industries, Elizabeth Macarthur Agricultural Institute, Menangle, NSW 2568 Australia

**Keywords:** CRISPR-Cas9, Guide RNA, Nanopore, Multiplexing, NCATS, Pathogen, Pest, Targeted

## Abstract

In agriculture, biosecurity, and human health, the rapid and accurate detection of pathogens and pests is crucial. Our study investigates the sensitivity and practicality of six guide RNA (gRNA) production methods for use in Nanopore Cas9-targeted sequencing (nCATS), focusing on their implications for multiplexed pathogen and pest detection. Each production method generated a library of eight gRNAs capable of excising ~ 1.6 kb fragments of the 5.8S_rRNA-ITS2-28S_rRNA regions of five economically significant wheat fungal pathogens. Through comparative analysis, we evaluated the efficacy of commercially synthesised and in-house *in vitro*-transcribed gRNAs, assessing their impact on sequencing enrichment outcomes. Our findings highlight differences amongst the methods in terms of gRNA yield, integrity, performance, and costs. Our best performing gRNA production method was able to successfully identify all target sequences across the 0.96 to 8.4 pg ranges we tested with coverage ranging from 66 to 2037X. This study highlights the challenges and opportunities in implementing nCATS for multiplexed pathogen and pest detection, offering insights into the development of cost-effective and reliable gRNA production strategies for nCATS.

**Supplementary Information:**

The online version contains supplementary material available at 10.1007/s12033-025-01510-9.

## Introduction

Rapid and accurate detection of pathogens and pests is crucial in agriculture, biosecurity, and human health [1, 2]. Traditional diagnostic methods, such as culturing organisms or PCR-based tests, often suffer from limitations, including targeting a limited number of pathogens and requiring laborious, time-consuming, and costly exclusion processes [3–5]. Consequently, it has been a long-standing goal within the scientific community to develop multiplexed assays capable of detecting numerous pathogens in a single test [6–8]. The combination of CRISPR-Cas9’s targeting specificity and the discriminatory power of next-generation sequencing (NGS) offers a promising avenue towards achieving this goal [9, 10].

Through iterative advancements, Cas9-targeted enrichment has emerged as a promising technique for generating targeted NGS libraries [10]. The first reported method, termed CATCH (Cas9-assisted targeting of chromosome segments), was used for isolating large DNA fragments by in-gel cleavage and separating the target region from the rest of the genomic DNA via pulse field gel electrophoresis [11, 12]. CATCH, coupled with Oxford Nanopore Technology (ONT) sequencing, provided a powerful tool for the real-time isolation of large DNA molecules. Initially developed to isolate the human breast cancer gene *BRCA1*, this method achieved 70X mean coverage, though only 1% of the reads were on target [13]. Despite its value, the enrichment process of CATCH is technically demanding and time-consuming.

CATCH was followed by an updated method, which started with the dephosphorylation of the genomic DNA, followed by targeted CRISPR–Cas9 cleavage, enabling the specific ligation of adapters to the phosphorylated termini at Cas9-cleaved sites [14, 15]. This technique allowed for high levels of multiplexing, enabling the detection of numerous target sequences within a single assay. In 2019, Quan et al. reported FLASH (Finding Low Abundance Sequences by Hybridization), an Illumina-based enrichment method that employed dephosphorylation and Cas9 cleavage, designed to target 3,624 clinically relevant antibiotic resistance genes in patient samples [14]. FLASH achieved five orders of magnitude enrichment of target genes and sub-attomolar gene detection. However, its reliance on the Illumina platform imposed constraints such as turnaround times, initial equipment costs, and demands for high-throughput consumables, limiting its suitability for routine testing [16].

In contrast, ONT sequencing offers real-time data acquisition, minimal initial investment costs, and greater flexibility in throughput options [17–19]. These advantages, as demonstrated in previous studies [17–22], make it a more viable sequencing technology for meeting the urgent and variable demands in diagnostic settings. In 2020, Gilpatrick *et al. *reported dephosphorylation-based Cas9-targeted enrichment in ONT library preparations, which they termed nCATS (Nanopore Cas9-targeted Sequencing) [15]. Their demonstration of nCATS for enriching disease-associated genomic loci, targeting 10 sites in the human genome, resulted in all loci yielding coverage greater than 400X. Since its development, nCATS has been applied to enrich target genomic regions in human, plant, and animal genomes [23–29].

Although nCATS is well established for genomic locus enrichment, its use in multiplex detection of low-abundance pathogens remains only sparsely explored. A notable example is Cottingham et al. (2025) [21], who used a commercially synthesised 62-guide panel to enrich *Klebsiella pneumoniae*multilocus sequence typing (MLST), transfer RNA and antimicrobial resistance loci. To bring nCATS into routine diagnostics we must define its sensitivity limits, sharpen guide-design rules and, critically, develop economical, high-fidelity gRNA production workflows. This includes assessment of the two available forms of Cas9 gRNAs: the conventional two-part guide RNAs, consisting of a crRNA and a tracrRNA (cr:tracrRNA), and engineered single-guide RNAs (sgRNAs), which combine crRNA and tracrRNA into a single molecule [30].

In this study, we examine the sensitivity and practicality of six gRNA production methods for nCATS, with a focus on their feasibility for multiplexed pathogen and pest detection. Each method produced a library of the same eight gRNAs capable of excising a ~ 1.6 kb fragment of the 5.8S_rRNA-ITS2-28S_rRNA region (referred to herein as only ITS2) in five economically significant wheat fungal pathogens: *Puccinia graminis* f. sp. *Tritici* (PGT), *Puccinia striiformis* f. sp. *Tritici* (PST), *Puccinia triticina* (PT), *Blumeria graminis* f. sp. *Tritici* (BGT), and *Zymoseptoria tritici* (ZT). These pathogens are responsible for stem rust, stripe rust, leaf rust, powdery mildew, and Septoria leaf blotch, respectively [31, 32].

## Methods

### DNA Sample Preparation

**Fig. 1:**
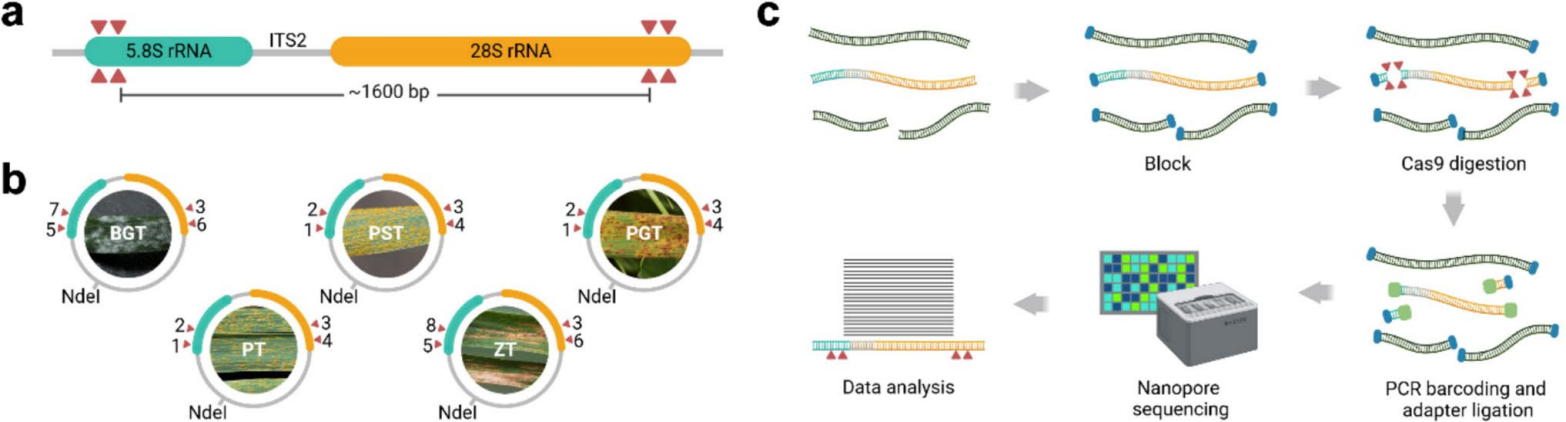
Workflow and guide RNA targets for Cas9-targeted sample enrichment. **a** gRNAs were designed to cleave each side of the 5.8S_rRNA-ITS2-28S_rRNA (ITS2) target region of five wheat fungal pathogens, to generate product fragments ranging in size from 1559 to 1694 bp. Two gRNAs were used at each Cas9 target site. **b** ITS2 target regions were commercially synthesised and cloned into pUC-GW-AMP plasmids with NdeI restriction sites in their backbone for linearising the plasmid. Each gRNA is presented by a red triangle and number. **c** Schematic of the Nanopore Cas9-targeted sequencing (nCATS) workflow. The process involves dephosphorylation of DNA ends to block their reactivity, followed by Cas9-targeted digestion to generate new phosphorylated ends flanking the target region. These phosphorylated ends undergo PCR-based barcoding and adapter ligation, before subsequent Nanopore sequencing and data analysis. Panel 1c was created with BioRender.com

Wheat genomic DNA (gDNA) was extracted from leaf tissue of Australian Hard variety wheat cultivated in autoclaved soil under controlled laboratory conditions using Plant Pro DNA extraction kit (Qiagen) following the manufacturer’s instructions. 5.8S_rRNA-ITS2-28S_rRNA (ITS2) region from the NCBI reference sequences of PGT, PST, PT, BGT, and ZT were synthesised (GeneWiz) and cloned into pUC-GW-AMP plasmid (Figs. 1a, b; S1 Table 1 for accession numbers and sequences). After plasmid amplification in One Shot TOP10 cells (Invitrogen), 1 µg of each plasmid was linearised with 10 U NdeI in 50 µl (37 °C, 1 h), heat inactivated at 65 °C for 20 min, gel purified (QIAquick Gel Extraction, Qiagen), and quantified with a Qubit fluorometer (Thermo Fisher). Following this, all five plasmid fragments were spiked into a single aliquot of wheat gDNA to make a master DNA sample, including linearised plasmid at concentrations of 28 pg PGT, 13.3 pg PT, 13 pg PST, 6 pg ZT, and 3.2 pg BGT per µg of wheat gDNA. These concentrations were not chosen to represent potential infection levels of the pathogen in crops, as no references to such levels were found in the literature. Instead, they were selected to test and push the sensitivity limits of the technique.

## gRNA Design and Production

A library of eight CRISPR RNAs (crRNAs) were designed using Geneious Prime’s CRISPR sites tool (Dotmatics) with default settings (NGG–SpCas9–3’ side), focusing on activity score [33] and specificity scoring [34] against the wheat reference genome (GCA_900519105.1). Refer to Fig. 1b for each gRNAs target site. crRNA sequences and DNA oligos for *in vitro* T7 transcription of gRNAs can be found in S1 Table 1.

**Fig. 2:**
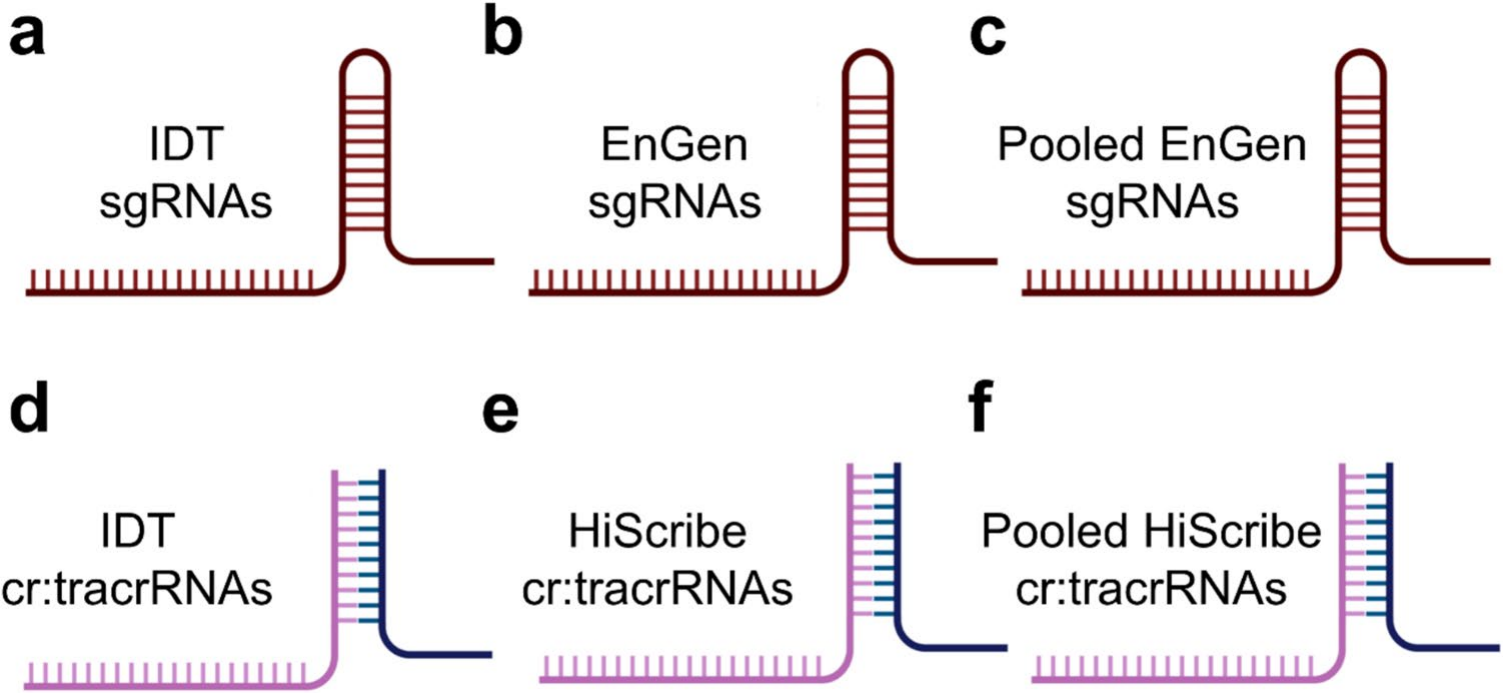
Guide RNA production methods. **a** IDT commercially manufactured single-guide RNAs (sgRNAs). The four in-house production methods were as follows: **b** sgRNAs individually transcribed with the EnGEN method before pooling equimolar. **c** sgRNAs transcribed as a pool with the EnGEN method in a single tube. **d** IDT commercially manufactured cr:tracrRNAs. **e** cr:tracrRNAs individually transcribed with the HiScribe method. **f** cr:tracrRNAs transcribed as a pool with the HiScribe method in a single tube. Created with BioRender.com

Six different gRNA production methods were assessed (Figs. 2a–f). We selected these methods due to their widespread kit availability and user-friendly protocols. Pooled transcription refers to the process of simultaneously transcribing multiple gRNAs from different DNA oligos in a single reaction tube.

Commercially manufactured crRNA, tracrRNA, and sgRNAs were synthesised by IDT. The remaining gRNA production methods were produced in-house.

crRNA and tracrRNAs were *in vitro* transcribed in duplicate reactions using the HiScribe T7 Quick High Yield RNA Synthesis Kit (NEB; HiScribe method), following the manufacturer’s instructions. For this, T7 primer and HiScribe DNA oligos were annealed in nuclease-free water at 10 μM by heating at 95 °C for 2 min and cooling to room temperature. In short, each 20-µl transcription reaction contained 1 µg of the annealed template to produce individual tracrRNAs, individual crRNAs, or pooled crRNAs. For pooled reactions, annealed templates were combined equimolarly to a total of 1 µg.

sgRNAs were transcribed in duplicate reactions using the EnGen sgRNA kit (NEB; EnGen method), following the manufacturer’s instructions, with either single or equimolar pooled EnGen DNA oligos being used as transcription templates.

All gRNA *in vitro* transcription reactions were purified using the Monarch RNA Cleanup (50 μg) Kit (NEB) and eluted into 50 μl of nuclease-free water. RNA molecules were quantified using the Qubit Broad Range RNA kit (Thermo Fisher), evaluated for integrity using a Bioanalyzer Small RNA kit (Agilent), and assessed for plasmid cleavage efficiency by complexing 10 pmol of a gRNA library with 10 pmol of HiFi Cas9 Nuclease V3 (IDT) in 30 μl 1X CutSmart Buffer (NEB) and digesting 300 ng of PST, BGT, or ZT plasmid before resolving products on Bioanalyzer using a DNA 7500 kit (Agilent). For *in vitro* plasmid digests, PST was used to represent plasmids PGT and PT, as all three plasmids share the same gRNA recognition sites in the same order and orientation (see Fig. 1b).

## Nanopore Cas9-Targeted Sequencing (nCATS)

All crRNA and tracrRNA reactions were duplexed in IDT duplex buffer by heating to 95ºC for 5 min and allowing cool to room temperature, resulting in a 10 μM solution. Ribonucleoprotein (RNP) complexes were assembled by mixing 10 pmol of one of the six gRNA libraries with 10 pmol of HiFi Cas9 Nuclease V3 (IDT) in 1X CutSmart Buffer (NEB), totalling a final volume of 30 μl at a concentration of 333 nM. RNP was complexed by incubating for 15 min at 25 °C before placing on ice.

Our approach for nCATS sample preparation was adapted from the methodology by Gilpatrick, Gilpatrick et al. 2020 (15). See Fig. 1c for schematic workflow. A DNA sample of 300 ng, comprising wheat gDNA and the ITS2-linearised plasmids, was made up to 90 μl in 1X CutSmart buffer. The DNA was then dephosphorylated using 6 μl of Quick CIP enzyme (NEB) for 10 min at 37 °C, followed by a 5 min at 80 °C to inactivate the enzyme. After cooling to room temperature, 28 μl from one of the six prepared RNP was added. Additionally, 1 μl of 10-mM dATP (NEB) and 1 μl of Taq DNA polymerase (NEB) were added for A-tailing. The sample was incubated at 37 °C for one hour for Cas9 cleavage, then at 72 °C for 10 min for A-tailing. Cas9 was inactivated by adding 1 μl (800 U) of Thermolabile Proteinase K (NEB) and incubating at 37 °C for 15 min, followed by 10 min at 80 °C. The sample was purified using AMPure XP magnetic beads (Beckman Coulter) and eluted into 22.5 μl of Milli-Q water. The nCATS samples were sequenced using the Ligation Sequencing Kit (SQK-LSK109; ONT) with the PCR Barcoding Expansion 1–12 kit (EXP-PBC001; ONT) with 30 rounds of PCR using LongAmp Taq DNA polymerase (NEB) as opposed to the 14 rounds in the manual. Barcoded samples were pooled in their entirety (without normalisation) and sequenced on a GridION sequencer with a MinION flow cell (R9.4.1; ONT), controlled by MinKNOW software (v.22.03.02; ONT).

Each gRNA library was used for a single nCATS enrichment. As the transcribed gRNA libraries were generated in duplicate (Transcription A and B), each gRNA production method was tested in duplicate.

## Conventional Nanopore Sequencing

As a comparator, we sequenced 300 ng of DNA master sample, containing wheat gDNA and the five linearised ITS2 plasmids without Cas9 target enrichment. Sample preparation was completed with ONT Ligation Sequencing Kit (SQK-LSK109) following the manufacturer’s instructions. DNA sequencing was carried out on a GridION sequencer with a MinION flow cell (R9.4.1; ONT). The sequencing was managed using MinKNOW software (v.22.10.7; ONT). The conventional ONT sample was sequenced as a singular run without replicates, utilising its own dedicated flow cell, to prevent the conventional sample from outcompeting the nCATS samples in pore occupancy.

## Data Analysis

Sequenced reads were basecalled using Guppy (v.6.0.6) in high-accuracy mode and filtered to retain only those with a quality score of ≥ 9 and length ≥ 1000 bp. Filtered data were then aligned to both the wheat genome (GCA_900519105.1) and target ITS2 regions (S1 Table 1) using minimap2 (v.2.22; [35]) with the parameters -a -k 15. Coverage metrics from the resulting SAM files were computed with Samtools Coverage (v1.9; mean depth and breadth) and Samtools Depth (v1.9; per-nucleotide depth) [36]. Depth profiles were visualised in Geneious Prime (2023.2.1), whilst statistics and graphs were prepared in Microsoft Excel (v2302) and GraphPad Prism (v9.3.0). Graphs in Figs. 4 and 5 were normalised to 2.2 × 10^7^ base calls (the mean number of base calls per barcoded sample), as opposed to the mean number of reads, due to the varied read length that occurs in ONT sequencing. Normalisation in Figs. 4 and 5 was performed to a single value (2.2 × 10⁷ base calls) rather than to each respective library, enabling direct comparisons of enrichment levels across all gRNA production methods and sequencing runs.

## Results

### Comparison of gRNA Yield and Integrity

We assessed six gRNA productions methods in duplicate to evaluate their suitability for multiplex nCATS for pathogen detection. There was significant variation in the yield of the gRNAs transcribed using the EnGEN and HiScribe methods, with the EnGEN transcriptions averaging of 693 ± 326.8 ng/μL, whilst HiScribe produced 1438.3 ± 1242.7 ng/μL (S1 Table 2). Although there was substantial variation in gRNA yield between gRNAs of different sequences, duplicate transcriptions of the same gRNA showed greater consistency across both methods, with an average standard deviation of ± 78.4 ng/μL, indicating a sequence-dependent transcriptional efficiency for both EnGEN and HiScribe methods.

**Fig. 3:**
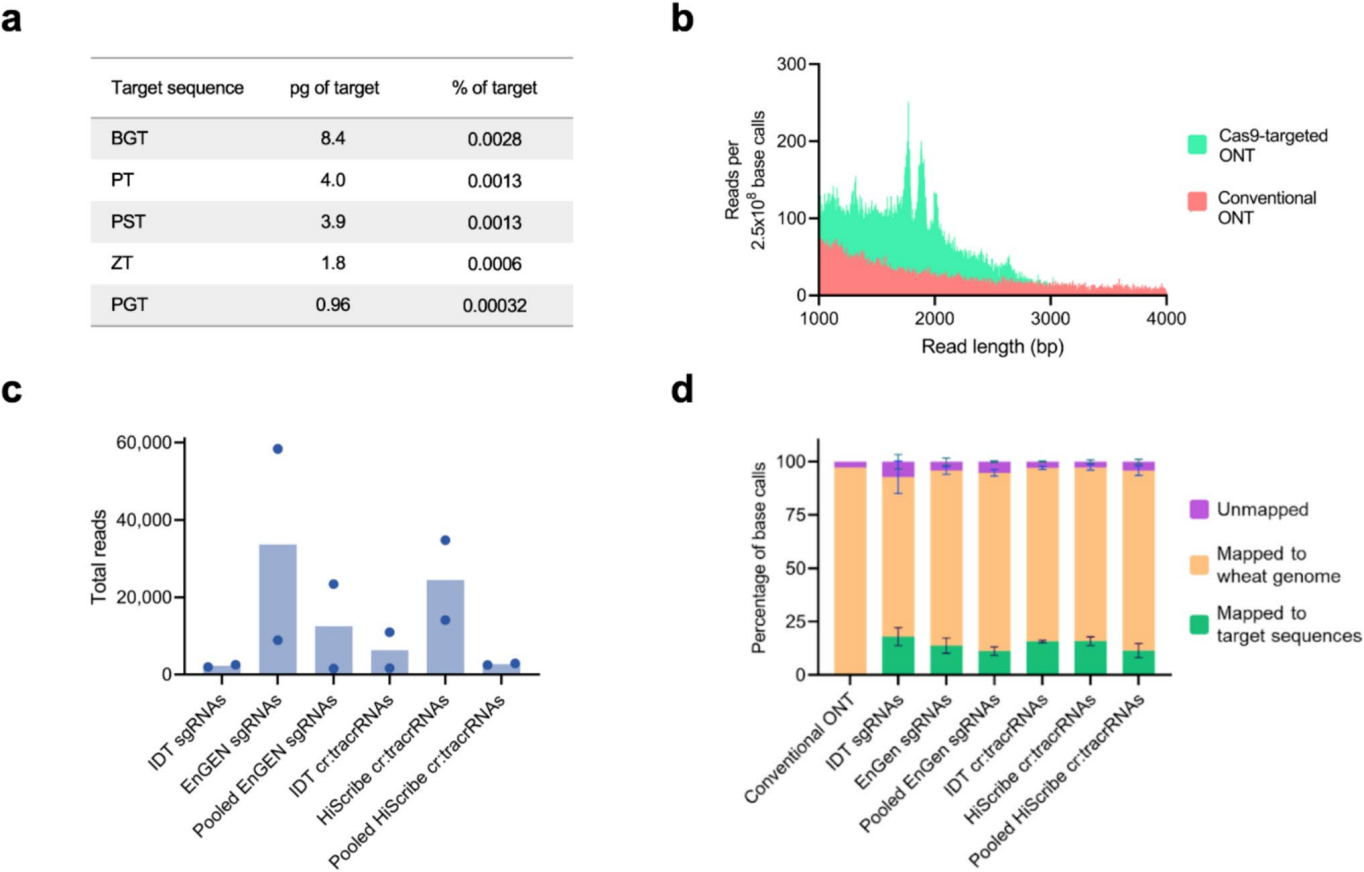
Multiplexed Nanopore Cas9-Targeted Sequences (nCATS). **a** Concentration of fungal pathogen ITS2 linearised plasmids in wheat genomic DNA. Each sample contained 300 ng of total DNA. **b** A comparison of sequencing read length abundance between Conventional ONT and nCATS-sequenced samples. **c** The mean number of reads collected from nCAT using the six different gRNA production methods. Coloured dots indicate individual sequencing run results. **d** The mean percentage of base calls that mapped using Minimap2 to the wheat genome, ITS2 target sequences, and unmapped for both Conventional ONT (n = 1) and the six difference gRNA production methods (n = 2). Error bars are standard deviation. ONT = Oxford Nanopore Technology sequencing

We evaluated gRNA integrity by automated electrophoresis (S1 Figs. 1 and 2) and assessed each gRNA library’s functionality by complexing them with Cas9 and testing their cleavage efficiency on PST, BGT, and ZT plasmids, which harbour recognition sites for all eight gRNAs. Automated electrophoresis confirmed the expected digestion patterns for each gRNA library (S1 Fig. 3), validating each library’s testing in subsequent multiplexed nCATS sequencing experiments.

### Performance of gRNA Libraries in nCATS Enrichment

For all nCATS sequencing experiments, each sample contained between 0.96 and 8.4 pg of a pathogen’s ITS2 linearised plasmid, representing 0.00032 to 0.0028% of the 300-ng DNA sample (Fig. 3a). Each sequencing run was prepared with a different gRNA library and barcoded. Collectively, the Cas9-targeted sequencing runs demonstrated distinct enrichment for reads with lengths ranging from 1.6 to 2 kb (Fig. 3b), corresponding to Cas9-enriched fragment sizes. In contrast, read length enrichment was absent in the conventional ONT-sequenced sample.

There was considerable variation in the total reads obtained from each nCATs barcoded sample, including substantial differences between runs prepared using the same gRNA production method (Fig. 3c). The lowest number of reads returned from a single-sequencing run after quality control was from an IDT cr:tracrRNA run with 1,696 reads. Conversely, samples prepared with individually transcribed EnGEN sgRNAs and HiScribe cr:tracrRNAs had the first and second highest number of total reads returned at 58,409 and 34,762 reads, respectively. The conventional ONT-sequenced sample, without nCATS enrichment, run on a dedicated flow cell, returned 1,310,243 reads after quality control (data not graphed).

### On-Target Enrichment and Coverage

Figure 3d represents a comparative mapping of both nCATS and conventional ONT data to the wheat reference genome and the five ITS2 pathogen target sequences. Using Minimap2, 0.05% of the total base calls from conventional ONT sequencing aligned to the target ITS2 sequences and 97.2% mapping to the wheat genome. All nCATS-sequenced samples had a substantially higher percentage of total base calls mapping to the pathogen ITS2 target sequences. The IDT sgRNA library had the highest average of on-target base calls, with a mean of 18 ± 6% mapping to the target sequences, whilst individual transcribed HiScribe cr:tracrRNA closely followed with 15.8 ± 2.9% on-target base calls. Pooled transcriptions had the lowest percentage on-target base calls, with EnGEN sgRNAs and HiScribe sgRNAs pooled libraries mapped, on average 11.2 ± 2.8% and 11.4 ± 4.7% of base calls to the target sequences, respectively.

**Fig. 4:**
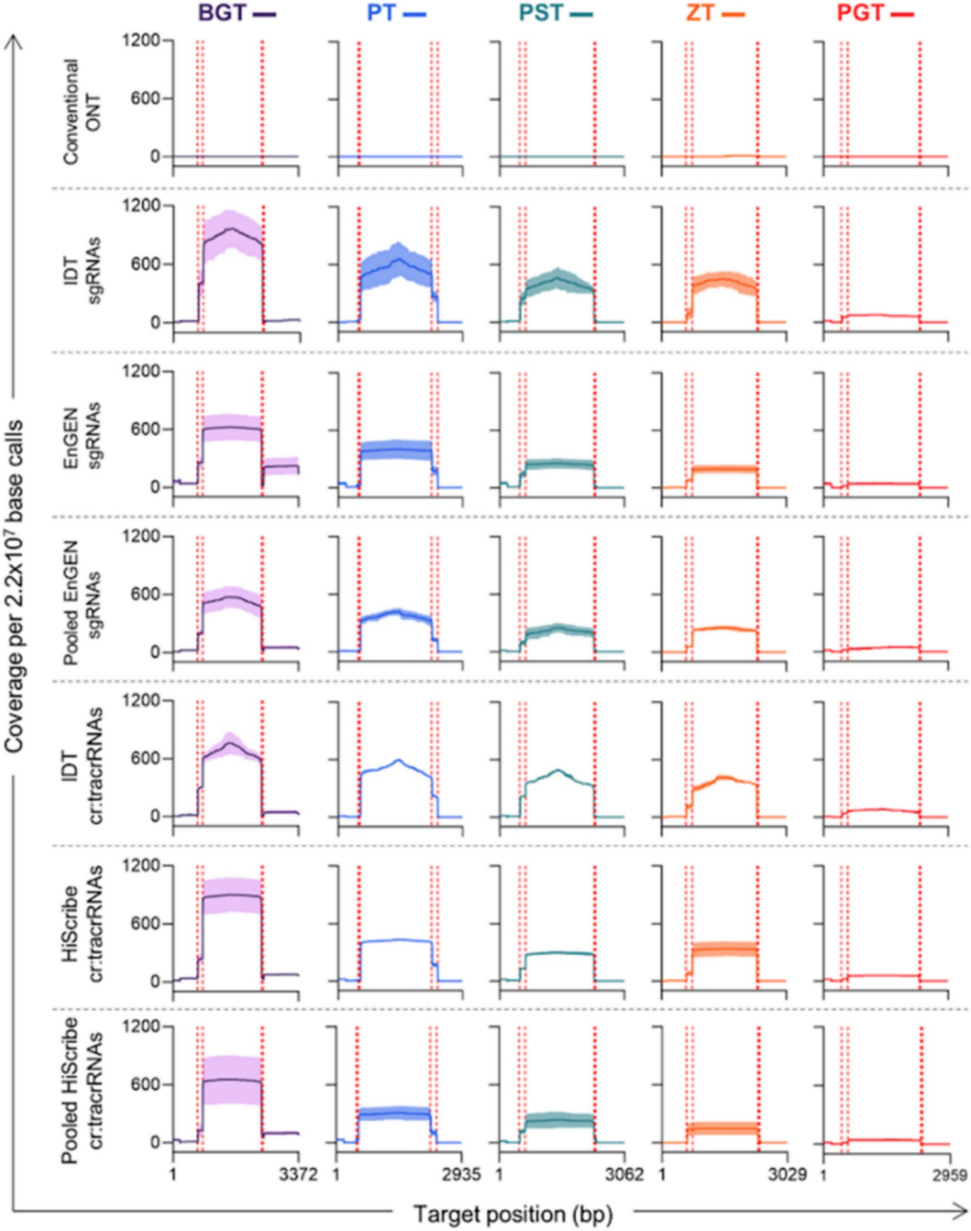
Mean coverage of each fungal pathogen ITS2 target sequence for conventional ONT and the six Cas9-targeted sample (n = 2) when normalised to mean number of base calls across libraries. Colored dots indicate individual sample results. ONT = Oxford Nanopore Technology sequencing

**Fig. 5:**
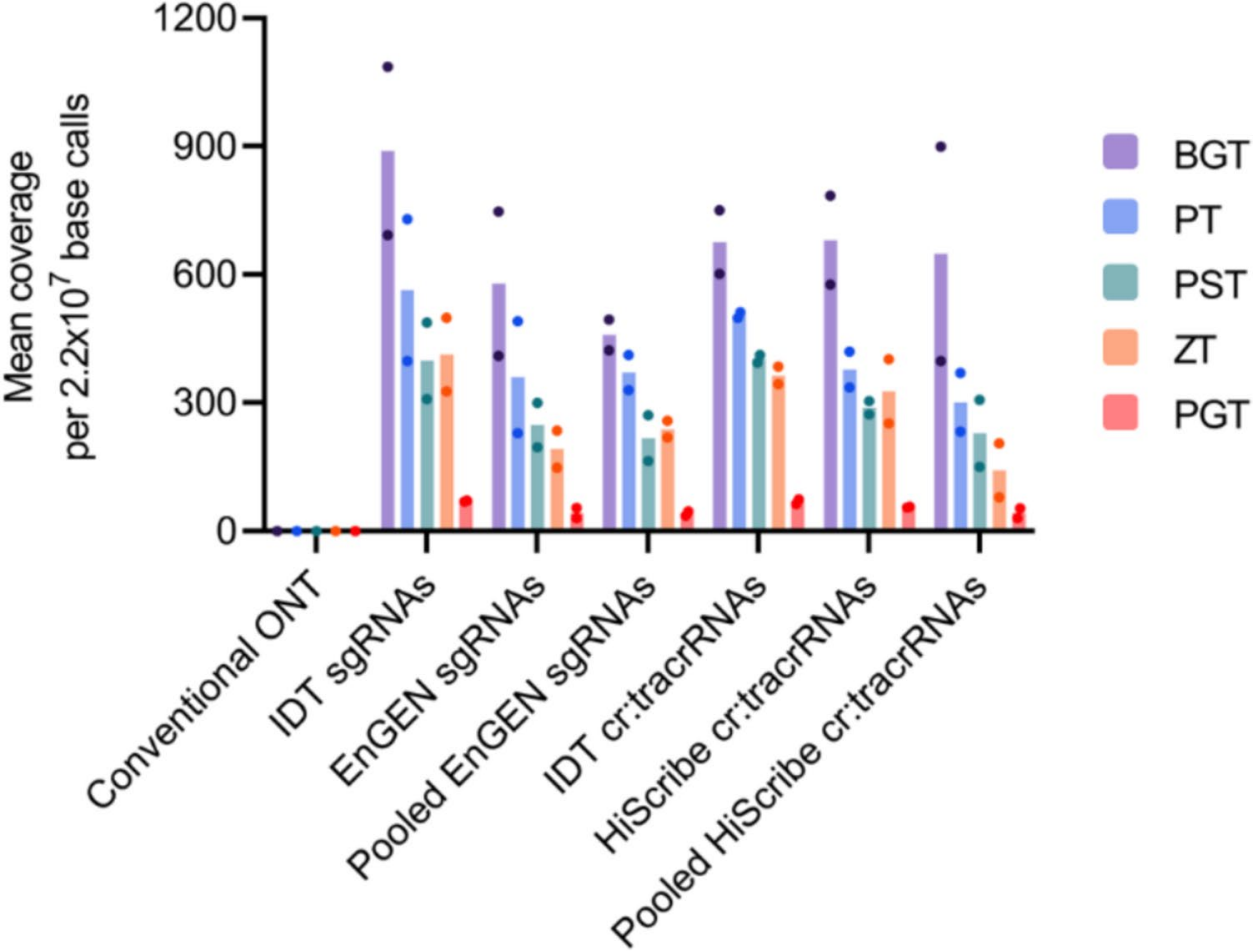
Mean coverage across the ITS2 target sequence for conventional Oxford Nanopore Technologies (ONT) and the six Cas9-targeted (nCATS) samples when normalised to mean number of base calls across libraries. The conventional ONT was a single-sequencing experiment whilst nCATS samples were run in duplicate, with shaded regions being standard deviation. Red-dashed lines indicated gRNA recognition sites

To compare enrichment levels, the results in Figs. 4 and 5 were normalised to 2.2 × 10^7^ base calls, representing the mean number of base calls per barcoded nCATS sample. After normalisation, Fig. 4 shows that conventional ONT sequencing had a mean coverage of less than one across the five ITS2 pathogen sequences per 2.2 × 10^7^ base calls. Amongst the nCATS samples, the IDT-synthesised gRNAs performed well. IDT sgRNAs had the highest mean coverage for BGT, PT, ZT, and PGT, whilst IDT cr:tracrRNAs had the highest mean coverage for PST. Pooled *in vitro-*transcribed libraries were not as well performing; of the three sgRNA production methods, the pooled EnGEN sgRNA was the worst performer for BGT, PST, and PGT, whilst of the cr:tracrRNA production methods, the pooled HiScribe cr:tracrRNA libraries was the worst performing across all five target sequences. Pooled transcriptions were suboptimal.

Figure 5 shows the mean normalised coverage across each ITS2 pathogen target region, highlighting a clear contrast between conventional ONT sequencing and nCATS. Conventional ONT sequencing scarcely mapped reads to the intended target sequences, with 0 or 1 read mapping per 2.2 × 10^7^ base calls. In contrast, the nCATS samples exhibited a marked increase in sequence coverage of the targets, initiating and concluding at the expected gRNA recognition sites. Notably, across all nCATS samples, the ZT target sequence had similar coverage to PST target, which had more than twice the concentration.

### Cost-Effectiveness and Diagnostic Potential

Based on mean yields of each production method, we calculated the costs associated with generating 1 nmol of a gRNA using each production method. Our cost estimations did not account for pooled gRNA transcriptions due to their relatively poor performance in this study, nor did they include labour and shipping costs. See S1 Table 3a for a summary of gRNA costs and S1 Tables 3b to e for cost calculations of each gRNA production method. Our calculations revealed that IDT sgRNA was the most expensive option, at AU$135 for the production of 1 nmol of a sgRNA, followed by IDT cr:tracrRNAs at AU$83 per nmol. Amongst the in-house transcription methods, EnGEN sgRNAs were priced at AU$52 per nmol, whilst HiScribe cr:tracrRNAs were the most economical option at AU$29 per nmol.

**Fig. 6:**
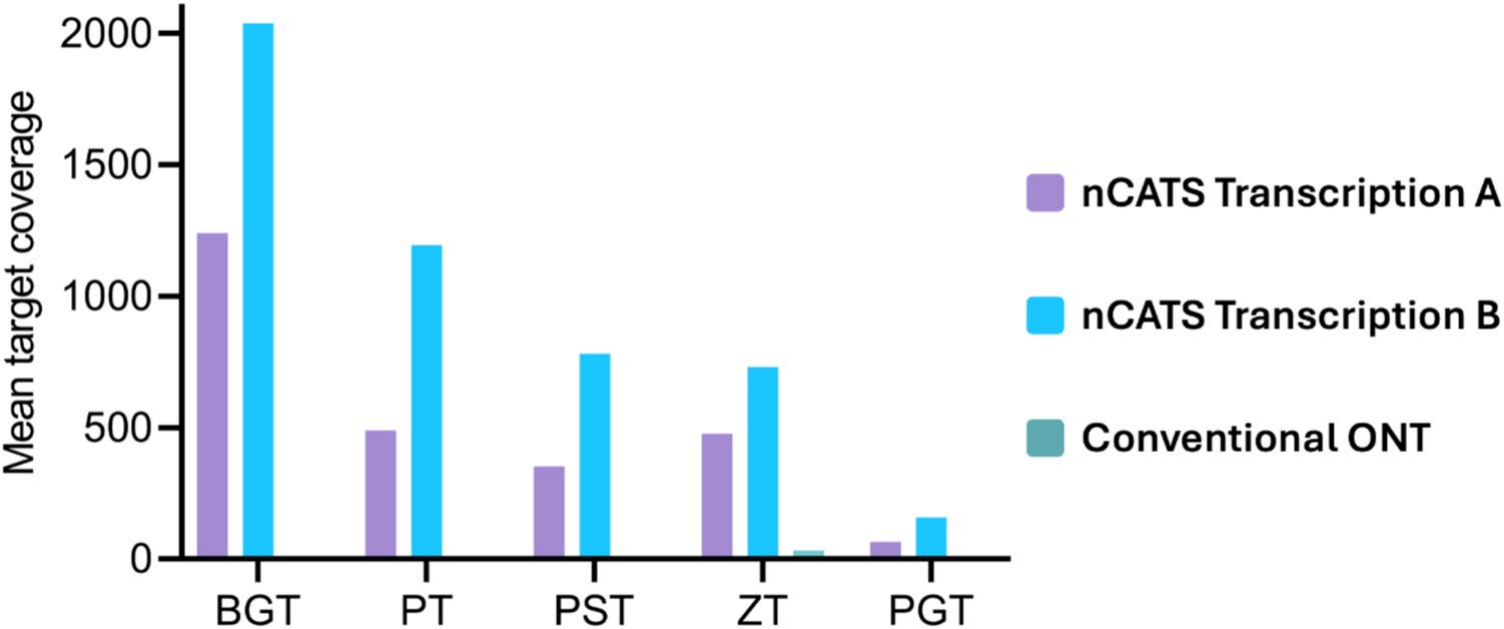
Mean target coverage for Cas9-targeted sequencing (nCATS) or conventional Oxford Nanopore Technology (ONT). 300 ng of master DNA sample was sequenced using either nCATS and HiScribe cr:tracrRNA gRNA library from Transcription A or B or conventional ONT sequencing. All data are presented without normalisation

Based on the performance, yield, cost, and practicality of production, we believe individually transcribed HiScribe cr:tracrRNAs were the best performing production method and further analysed the results from these runs. In the absence of data normalisation, Fig. 6 illustrates the mean coverage for HiScribe cr:tracrRNA prepared samples ranged from 66X for PGT to 2037X for BGT. This level of coverage facilitated the identification of all five target species with a maximum of four nucleotide ambiguities between the generated consensus sequences and the reference sequence (refer to S1 Tables 4 to 8 for consensus sequence alignments to ITS2 references). Notably, these ambiguities were only found in homopolymeric stretches of four nucleotides or more, a common artefact of ONT sequencing. In contrast, the 4.3 gigabases of data from the conventional ONT sequencing exhibited fragmented mean on-target coverage ranging from 0.6 to 33X, which was insufficient coverage to accurately identify the five target species.

## Discussion

Our study highlights the potential of nCATS for multiplexed detection of low-abundance nucleic acids associated with pathogens and pests. Amongst the four in-house gRNA production methods that we tested, we observed significant variability in the yield and integrity of transcribed gRNAs produced with EnGEN or HiScribe methods (S1 Table 2). Based on the observed variations in yield between different gRNA template sequences, we suggest that gRNA sequences can be optimised in design to improve T7 *in vitro *transcription efficiency. Known parameters include template GC content, secondary structures, and poly-A and poly-T sequences [37]. We think that there is scope for gRNA design tools to incorporate algorithm options to select gRNAs optimised for T7 *in vitro* transcription.

We found that pooled transcribed HiScribe cr:tracrRNAs and EnGen sgRNAs were the most practical and cost-effective to generate, with the entire gRNA library being produced in a single reaction. However, pooled transcription reactions inevitably lead to varying concentrations of each gRNA due to differences in template transcription efficiency. Since quality control cannot be performed on each gRNA within a pooled transcription, this is undesirable in diagnostic settings. In contrast, individually transcribed gRNAs, which are equimolar pooled following transcription, enable quality control of each gRNA but require robotic liquid handling equipment for practical scalability.

Within each nCATS sample preparation, there are three magnetic bead clean-up steps of the DNA sample. In this study, we performed these steps manually, which may have contributed to variations in the total reads returned. Standardising nCATS sample preparations with robotic liquid handlers, including bead clean-up steps, could improve the consistency of the total reads returned. However, variation in total reads returned from nCATS is not unique to this study and several other projects have reported similar outcomes [15, 26, 27, 29, 38]. Protocol optimisation to improve yield consistency will help advance nCATS from an emerging technique to a routine diagnostic technique.

In evaluating gRNA performance, we found that commercially synthesised IDT crRNAs or sgRNAs consistently exhibited low total read returns, but high on-target read percentages (Figs. 3c, d). Amongst the four *in vitro*-transcribed methods, individually transcribed HiScribe cr:tracrRNA and EnGEN sgRNAs yielded the highest mean total reads and demonstrated high levels of on-target enrichment at 15.8 ± 2.9 and 13.8 ± 5%, respectively. Pooled EnGEN and HiScribe gRNA transcriptions returned significantly fewer total reads and less enrichment than their individually transcribed counterparts.

Considering performance, yield and cost, we concluded that individual transcription of cr:tracrRNAs using the HiScribe method is the most effective of the tested methods for gRNA library production for nCATS. This method produced cost-effective gRNAs (AU$29 per nmol), the second highest mean number of total reads, exhibited high levels of on-target enrichment (15.8 ± 2.9%), and identified all five target sequences in both duplicate sequencing runs (S1 Tables 4 to 8), with enrichment ranging from 66 to 2027X across the 0.96 to 8.4 pg target concentrations (Fig. 6). Our selected HiScribe method adopts the principal components of Gilpatrick *et al.* 2023 [35] gRNA production method but transcribes each gRNA individually rather than pooling them.

Potential methods to improve assay sensitivity beyond what was reported in this study include increasing the number of PCR cycles during PCR-based barcoding and optimising the type of polymerase used [39, 40]. The ONT PCR barcoding kit utilises universal primer sequences. In this study, we increased the PCR barcoding cycles from the ONT-recommended 14 cycles to 30 cycles using LongAmp Taq DNA polymerase (NEB) to amplify the target ITS2 fragments. Increasing the number of cycles post-adapter ligation beyond 30 cycles, along with optimising the polymerase type (currently designed for long fragments), could further enhance assay sensitivity for the shorter fragment lengths used in this study.

An important insight from this study and our recent study, McFarlane et al. 2024 (26)was observed. The role that gRNA directionality plays in influencing enrichment levels, advocating for the orientation of gRNAs with protospacer adjacent motif (PAM) sites directly adjacent the target enrichment region. Notably, despite PST having more than twice the concentration of ZT, both exhibited similar enrichment levels across gRNA preparations, with comparable gRNA cleavage efficiencies, as demonstrated by *in vitro *cleavage assays in S1 Fig. 3. This suggests that the observed results were not due to differences in gRNA efficiency. ZT inner most gRNAs, had PAM sequences orientated directly adjacent to both ends of the enriched fragments. However, PST and all other gRNAs had PAM sites orientated so that either one or both of the inner most gRNAs had PAM sites adjacent to the enrichment region (S1 Fig. 4 shows gRNA directionalities). This bias in orientation has been observed in other nCATS studies, and is often linked to Cas9 remaining bound to the non-PAM side of the target DNA after cleavage, preventing ONT sequencing adapter ligation [15, 25, 41].

Within this study, we hypothesised that treatment with Proteinase K followed by heat treatment at 80 °C would dislodge bound Cas9, enabling adapter ligation at cleavage sites [42–44]. However, our findings in corroboration with those of Stephenson, Raper and Suo [45] suggest that post-cleavage 5′ → 3′ degradation by Cas9 on the non-PAM side would impede adapter ligation, rather than the enzyme remaining bound post-cleavage. This preferential generation of staggered-ended DNA products on the non-PAM side is likely responsible for inhibiting adapter ligation. We recommend that all gRNAs should be oriented with PAM sites adjacent to the target enrichment region to further improve enrichment levels.

Conventional ONT sequencing demonstrated markedly low target coverage, significantly below the anticipated 0.00032 to 0.0028% proportion of target DNA in the sample. This limited coverage is likely due to the presence of long wheat genomic DNA strands, which occupy Nanopores for extended periods, thereby reducing the availability of pores for sequencing target DNA. Given the inherent variability in read lengths associated with Nanopore sequencing, we elected to compare target and non-target DNA based on base calls rather than on a per-read basis. This methodology provides a more accurate assessment of the relative sequencing depth of target versus non-target DNA. In contrast, evaluating on-target to non-target DNA on a per-read basis could increase on-target mapping percentages for conventional ONT sequencing.

As diagnosticians increasingly adopt high-throughput methods capable of simultaneously detecting numerous pathogens and pests in a single sample, nCATS emerges as a promising technique. nCATS benefits from real-time data acquisition, minimal initial investment costs, and high flexibility in both multiplexing capabilities and sample throughput [15, 17, 18]. Also, it significantly reduces computational storage and performance requirements compared to conventional NGS diagnosis of low-abundance sequences. Of the six gRNA production methods tested in this project, we found that individually transcribed cr:tracrRNA using the HiScribe method offer a cost-effective, practical, and high-performing option for gRNA production for nCATS.

Recent nCATS workflows for pathogen profiling [21] and antimicrobial-resistant surveillance [20] relied on 62 and 10 commercially synthesised gRNAs, respectively. Using the HiScribe in-house transcription method described here would cut guide costs by roughly two-thirds, making large-panel assays affordable for both research and routine diagnostics. We recommend that future development of multiplexed nCATS should focus on optimising gRNA design tools for T7 *in vitro *transcription efficiency and gRNA directionality to enhance enrichment. Additionally, standardising nCATS sample preparations using robotic liquid handlers may help achieve more consistent sequencing yields.

Despite these advantages, our study has several limitations. We assessed only eight gRNAs targeting ~ 1.6 kb ITS2 fragments from five wheat-associated fungi, so performance may differ for longer targets, other genomic contexts, or highly multiplexed panels. We benchmarked only a subset of transcription methods and reagents, and a single-sequencing platform (MinION R9.4.1), leaving alternative chemistries, flow cells, and bioinformatic pipelines unexplored. Finally, the cost analysis reflects current Australian reagent prices and may shift with scale, supplier discounts, or regional pricing.

Our next phase will test the workflow on real-world samples and benchmark it against current gold-standard diagnostics, both to confirm sensitivity and specificity for fungal crop pathogens and to explore its applicability to other agricultural pests and pathogens.

## Supplementary Information

Below is the link to the electronic supplementary material.

Supplementary file1 (PDF 1876 kb)

## Data Availability

The data generated in this study can be found under the NCBI BioProject PRJNA1064613, with accession numbers SRR27544979 to SRR27544991.
