# Supplementary material for: Optimising Guide RNA Production for Multiplexed Cas9-Targeted Nanopore Sequencing to Detect Pathogens
Source: Mol Biotechnol. 2025 Sep 7;68(5):2399–409. doi: 10.1007/s12033-025-01510-9 (PMC13212634; doi:10.1007/s12033-025-01510-9)
Supplement: Supplementary file 1 — Supplementary file1 (PDF 1876 kb) [file 12033_2025_1510_MOESM1_ESM.pdf]

S1_File: Guide RNA production for multiplexed Cas9-targeted Nanopore sequencing

Authors: Gus R McFarlane, Kim Whitaker, Krista L Plett, Brendon O'Rourke and Daniel R Bogema

S1 Table 1. Nucleic acid name, sequence and application.

Nucleic acid name	Sequence	Application
crRNA_1	TTATAAATAACTTTTAACAA	crRNA sequenced for IDT crRNA and sgRNAs
crRNA_2	GGGTTTCATGACACTCAAAC	crRNA sequenced for IDT crRNA and sgRNAs
crRNA_3	CCACCAAGATCTGCACTAG	crRNA sequenced for IDT crRNA and sgRNAs
crRNA_4	GAGAACCTTGAAGACTGAAG	crRNA sequenced for IDT crRNA and sgRNAs
crRNA_5	ACTTTCAACAACGGATCTCT	crRNA sequenced for IDT crRNA and sgRNAs
crRNA_6	AGAACTTTGAGGACTGAAGT	crRNA sequenced for IDT crRNA and sgRNAs
crRNA_7	GTTGTTACGGACGCTCGAAC	crRNA sequenced for IDT crRNA and sgRNAs
crRNA_8	TTACACCACTCCAGCCTCGC	crRNA sequenced for IDT crRNA and sgRNAs
HiScribe_DNA_1	CAAAACAGCATAGCTCTAAAACTTGTTAAAAGTTATTTATAACTATAGTGAGTCGTATTA	DNA oligo for HiScribe transcription
HiScribe_DNA_2	CAAAACAGCATAGCTCTAAAACGTTTGAGTGTCATGAAACCCCTATAGTGAGTCGTATTA	DNA oligo for HiScribe transcription
HiScribe_DNA_3	CAAAACAGCATAGCTCTAAAACCTAGTGCAGATCTTGGTGGCTATAGTGAGTCGTATTA	DNA oligo for HiScribe transcription
HiScribe_DNA_4	CAAAACAGCATAGCTCTAAAACCTTCAGTCTTCAAGGTTCTCCTATAGTGAGTCGTATTA	DNA oligo for HiScribe transcription
HiScribe_DNA_5	CAAAACAGCATAGCTCTAAAACAGAGATCCGTTGTTGAAAGTCTATAGTGAGTCGTATTA	DNA oligo for HiScribe transcription
HiScribe_DNA_6	CAAAACAGCATAGCTCTAAAACACTTCAGTCCTCAAAGTTCTCTATAGTGAGTCGTATTA	DNA oligo for HiScribe transcription
HiScribe_DNA_7	CAAAACAGCATAGCTCTAAAACGTTCGAGCGTCCGTAACAACCTATAGTGAGTCGTATTA	DNA oligo for HiScribe transcription
HiScribe_DNA_8	CAAAACAGCATAGCTCTAAAACGCGAGGCTGGAGTGGTGTAACTATAGTGAGTCGTATTA	DNA oligo for HiScribe transcription
HiScribe_DNA_tracrRNA	AAAAAGCACCGACTCGGTGCCACTTTTTCAAGTTGATAACGGACTAGCCTTATTTTAACTTGCTATGCTGTCCTA TAGTGAGTCGTATTA	DNA oligo for HiScribe transcription
T7_primer	TAATACGACTCACTATAG	T7 primer for HiScribe transcriotion
EnGEN_DNA_1	TTCTAATACGACTCACTATAGTTATAAATAACTTTTAACAAGTTTTAGAGCTAGA	DNA oligo for EnGEN sgRNA kit
EnGEN_DNA_2	TTCTAATACGACTCACTATAGGGTTTCATGACACTCAAACGTTTTAGAGCTAGA	DNA oligo for EnGEN sgRNA kit
EnGEN_DNA_3	TTCTAATACGACTCACTATAGCCACCAAGATCTGCACTAGGTTTTAGAGCTAGA	DNA oligo for EnGEN sgRNA kit
EnGEN_DNA_4	TTCTAATACGACTCACTATAGAGAACCTTGAAGACTGAAGGTTTTAGAGCTAGA	DNA oligo for EnGEN sgRNA kit
EnGEN_DNA_5	TTCTAATACGACTCACTATAGACTTTCAACAACGGATCTCTGTTTTAGAGCTAGA	DNA oligo for EnGEN sgRNA kit
EnGEN_DNA_6	TTCTAATACGACTCACTATAGAGAACTTTGAGGACTGAAGTGTTTTAGAGCTAGA	DNA oligo for EnGEN sgRNA kit
EnGEN_DNA_7	TTCTAATACGACTCACTATAGTTGTTACGGACGCTCGAACGTTTTAGAGCTAGA	DNA oligo for EnGEN sgRNA kit
EnGEN_DNA_8	TTCTAATACGACTCACTATAGTTACACCACTCCAGCCTCGCGTTTTAGAGCTAGA	DNA oligo for EnGEN sgRNA kit

Nucleic acid name	Sequence	Application
BGT_96224_28S	ATTTAGAGGAAGTAAAAGTCGTAACAAGGTTTCCGTAGGTGAACCTGCGGAAGGATCATTACAGAGCGTGAAGCT<br>ATGCGGAACTTCGTTTCGTGTAGTTGACCCTCCACCCGTGTCGATTATCTCATGTTGCTTTGGCGGATCGGGCTT<br>GCCCGCGCCCACTTTGTGGGGATATCCGCCAGGGAAGACCAAAACTCTTGTTATCAGTGATGTCTGAGGATGAT<br>ATATAATCATGAAAACTTTCAACAACGGATCTCTTGGCTCTGGCATCGATGAAGAACGCAGCGAAATGCGATAAG<br>TAATGTGAATTGCAGAATTTAGTGAATCATCGAATCTTTGAACGCACATTGCGCCCCTGGGAATTCCCAGGGGCA<br>TGCCTGTTCGAGCGTCCGTAACAACCTCTCAAGCCTGGCTTGGTATTGGGACTCGCTGCCTCGGTGGTGGTCCTC<br>AAAAGCAGTGGCGGGCCCATGTAACTCTCCGCGTAGTAATACATCTCGCGACAGAGAAGCAGCGGGACTTGCCAA<br>AACTCCTTAATTGCTCAGGTTGACCTCGAATCAGGTAGGGATACCCGCTGAACTTAAGCATATCAATAAGCGGAG<br>GAAAAGAAACCAACAGGGATTACCTCAGTAACGGCGAGTGAAGCGGTAACAGCTCAAATTTGAAATCTGGTCCGT<br>TTACGGGCCCGAGTTGTAATTTGTAGAAGATGCTTTGGTGACGAGTCCGGCCTAAGTTCCTTGGAACAGGACGTC<br>ATAGAGGGTGAGAACCCGTATGCGGCCGGGTGCTCGATGCTATGTAAAGCTCTTTCGACGAGTCGAGTTGTTTGG<br>GAATGCAGCTCAAAATGGGTGGTAAATTTCATCTAAAGCTAAATATTGGCCAGAGACCGATAGCGCACAAGTAGA<br>GTGATCGAAAGATGAAAAGCACTTTGGAAAGAGAGTTAAACAGTACGTGAAATTGTTGAAAGGGAAGCGCTTGCA<br>ACCAGACTTGGGCACTGTGGATCATCCGAGGTTCTCCTCGGTGCACTCGGCAGTGCGCAGGCCAGCATCAGTTTG<br>GGTGGTTGGATAAAGACTTGTGGAATGTAGCTCCTTTCGGGAGTGTTATAGCCACAGGTGCCATGCGACCAACCC<br>GGACTGAGGACCGCGCTTCGGCTAGGATGCTGGCGTAATGGTTGTAAGCGACCCGTCTTGAAACACGGACCAAGG<br>AGTCTAACATCTATGCGAGTGTTTGGGTGTTAAACCCATACGCGGAATGAAAGTGAACGTAGGTGAGAACCCATA<br>AGGGGGCATCATCGACCGATCCGGATGTCTTCGGATGGATTTGAGTAAGAGCATAGCTGTTGGGACCCGAAAGAT<br>GGTGAACTATGCCTGAATAGGGTGAAGCCAGAGGAAACTCTGGTGGAGGCTCGCAGCGGTTCTGACGTGCAAATC<br>GATCGTCAAATTTGGGTATAGGGGCGAAAGACTAATCGAACCATCTAGTAGCTGGTTCCTGCCGAAGTTTCCCTC<br>AGGATAGCAGTGTTGAATTCAGTTTTATGAGGTAAAGCGAATGATTAGAGGCCTTGGGGTTGAAACAACCTTAAC<br>CTATTCTCAAACTTTAAATATGTAAGAAGTCCTTGTTACTTAATTGAACGTGGACAGCCGAATGTACCAACACTA<br>GTGGGCCATTTTTGGTAAGCAGAACTGGCGATGCGGGATGAACCGAACGTGAAGTTAAGGTGCCGGAATACACGC<br>TCATCAGACACCACAAAAGGTGTTAGTTCATCTAGACAGCAGGACGGTGGCCATGGAAGTCGGAATCCGCTAAGG<br>AGTGTGTAACAACTCACCTGCCGAATGAACTAGCCCTGAAAATGGATGGCGCTTAAGCGTGTTACCCATACTTCA<br>CCGCCCGGGTAGAAACGATGCCCGGGCGAGTAGGCAGGCGTGGAGGTCAGTGACGAAGCCTTGGGAGTGATCCCG<br>GGTCGAACGGCCTCTAGTGCAGATCTTGGTGGTAGTAGCAAATACTCAAATGAGAACTTTGAGGACTGAAGTGGG<br>GAA	5.8S-ITS2-28S synthesised sequence for Blumeria graminis f. sp. Tritici (BGT) from GenBank assembly: GCA_000418435. Synthesised by GeneWiz.
PGT_CRL_28S	TTTAATTTATAAATAACTTTTAACAATGGATCTCTAGGCTCTCACATCGATGAAGAACACAGTGAAATGTGATAA<br>GTAATGTGAATTGCAGAAATTCAGTGAATCATCGAATCCTTTGAACGCATCTTGCACCTTTTGGTATTCCAAAAA<br>GGTACACCTGTTTGAGTGTCATGAAACCCTCTCATTAAACATTTTTTTAATAATTTGTTTATTGGATGTTGAGTG<br>TTGCTATAATTATAGCTCACTTTAAATATATAAGTCACTTTTCAAATAAGTTGGATTGACTTGGTGTAATAATTT<br>TATCATCACATCAAGGAAAGTAGTCAAACACTTGCCATTTTTTTTGTAACAAGAGACTCCTAAAAACCCAATATC<br>TATTTTTTTAAGACCTCAAATCAGGTGGGACTACCCGCTGAACTTAAGCATATCAATAAGCGGAGGAAAAGAAAC<br>TAACAAGGATTCCCCTAGTAACGGCGAGTGAAGAGGGAAAAGCCCAAATTTGTAATCTGGCTCTTTCAGAGTCCG<br>AGTTGTAATTTTAAGAACTGTTTTCAGTGCTGGACCATGTATAAGTCTGTTGAAAAGCAGCATCATTGAGGGTGA<br>TAATCCCGTTTATGATATGGACTACCAGTGCATTATGATACAGTCTCTAAGAGTCGAGTTGTTTGGGAATGCAGC<br>TCAAAGTGGGTGGTAAATTCCATCTAAGGCTAAATATAGGTGAGAGACCGATAGCAAACAAGTACCGTGAGGGAA<br>AGATGAAAAGAACTTTGGAAAGAGAGTTAACAGTACGTGAAATTGTTAAAAGGGAAACACTTGAAGTTAGACTTG<br>TTATTATTAGTTCAACCTTTTTATTAAGGGAGTATTCTAATGATTAACAGACCAACATCAATTTTTGAGTGTTGG<br>AGAAGGGTTTAAGGAAATGTAGCAGTCTTTGGCTGTGTTATAGTCCTGAGCTTTGATACAATGCTTAAGATTGAG<br>GAAGGCAGTAAGCGCAATTTATTGTGTGGAACAAATTAATGTTCTTCTTACTGAGGATGTTGGTGTAATAGCTTT<br>AAATGACCCGTCTTGAAACACGGACCAAGGAGTCTAACATGCTTGCAAGTATTTGGGTGCTTGAAACCCTTATGC<br>GTAATGAAAGTAAATGTAAATGGGATCTGTCAAAAGTGCACCATTGACCAGTCCAGATTATTTATATGATGGTAC<br>TGAGTAAGAGCAAGTATGTTGGGACCCGAAAGATGGTGAACTATGCCTGAATAGGGGTGAAGCCAGAGGAAACTC<br>TGGTGGAAGCTCGTAGCGGTTCTGACGTGCAAATCGATCGTCAAATTTGGGTATAGGGGCGAAAGACTAATCGAA<br>CCATCTAGTAGCTGGTTCCTGCCGAAGTTTCCCTCAGGATAGCAAAGACTCGTATCAGTTTTATGAGGTAAAGCG<br>AATGATTAGAGGCCTTGGGGATGAAACATCCTTAACCTATTCTCAAACTTTAAATATGTAAGACGCTCCTGTTTC<br>TTAATTGAACGTGGGCATGTGAATGAGAGTCTTTAGTGGGCCATTTTTGGTAAGCAGAACTGGCGATGCGGGATG<br>AACCGAACGTGAGGTTAAGGTGCCGGAATATACACTCATCAGACACTACAAAAGGTGTTAGTTCATCTAGACAGC<br>CGCACGGTGGCCATGGAAGTCGGAATCCGCTAAGGAGTGTGTAACAACTCAACGGCCGAATGAACTAGCCCTGAA<br>AATGGATGGCGCTTAAGTGTATTACCCATACCTCACCATTAATATTGTTTTTCATTACATATTAATGAGTAGGCA<br>GGCGTGGAGGTTATGTAGCGAAGCCTTGGCAGTGATGCTGGGTGGAACAGCCTCTAGTGCAGATCTTGGTGGAAG<br>TAGCAAATATTCAAGTGAGAACCTTGAAGACTGAAGTGGGGAAG	5.8S-ITS2-28S synthesised sequence for Puccinia graminis f. sp. Tritici (PGT)from GenBank assembly: GCA_000149925.1. Synthesised by GeneWiz.
PST_38S102_28S	ACCCTCCCTTTTTTTTTTTATTAAAAATTACACAAAACACAAGTTTAAATGAATGTAACCAAACCTTTAATTATA<br>AATAACTTTTAACAATGGATCTCTAGGCTCTCACATCGATGAAGAACACAGTGAAATGTGATAAGTAATGTGAAT<br>TGCAGAATTCAGTGAATCATCGAATCTTTGAACGCACCTTGCGCCTTTTGGTATTCCAAAAGGCACACCTGTTTG<br>AGTGTCATGAAACCCTCTCATTAAATAATTTTGATTAATTATTTTCAATGGATGTTGAGTGCTGCTGTAATTAGC<br>TCACTTTAAATATATAAGTCACTTTTCTATAAGTTGGATTGACTTGGTGTAATAATTTTATCATCACATCAAGGA<br>TTGTAGCAATACTGCCATCTTATTTAAGGGAGACTCCTAAAAACCCAATTTTAACCTTAAGACCTCAAATCAGGT<br>GGGACTACCCGCTGAACTTAAGCATATCAATAAGCGGAGGAAAAGAAACTAACAAGGATTCCCCTAGTAACGGCG<br>AGTGAAGAGGGAAAAGCCCAAATTTGTAATCTGGCTCTTTCAGAGTCCGAGTTGTAATTTTGAGAACTGTTTTCA<br>GTGCTGGACCATGTATAAGTCTGTTGAAAAGCAGCATCATTGAGGGTGATAATCCCGTTCATGATATGGACTACC<br>AGTGCATTATGATACAGTCTCTAAGAGTCGAGTTGTTTGGGAATGCAGCTCAAAGTGGGTGGTAAATTCCATCTA<br>AGGCTAAATATAGGTGAGAGACCGATAGCAAACAAGTACCGTGAGGGAAAGATGAAAAGAACTTTGGAAAGAGAG<br>TTAACAGTACGTGAAATTGTTAAAAGGGAAACACTTGAAGTTAGACTTGTTATTATTAGTTCAACCTTTTTGACG<br>AGGGAGTATTCTAATGATTAACAGACCAACATCAATTTTTGGGTGTTGGAGAAGGGTTTAAGGAAATGTAGCAGT<br>CTCTGACTGTGTTATAGTCCTGAGCTTTGATACAATGCTTAAGATTGAGGAAGGCAGTAAGCGCAATTTATTGTG<br>TGGAACAAATTAATGTTCTTCTTACTGAGGATGTTGGTGTAATAGCTTTAAATGACCCGTCTTGAAACACGGACC<br>AAGGAGTCTAACATGCTTGCAAGTATTTGGGTGCTTGAAACCCTTATGCGTAATGAAAGTAAATGTAAATGGGAT<br>CTGTTAAAAGTGCACCATTGACCAGTCCAGATTATTTATATGATGGTACTGAGTAAGAGCAAGTATGTTGGGACC<br>CGAAAGATGGTGAACTATGCCTGAATAGGGTGAAGCCAGAGGAAACTCTGGTGGAAGCTCGTAGCGGTTCTGACG<br>TGCAAATCGATCGTCAAATTTGGGTATAGGGGCGAAAGACTAATCGAACCATCTAGTAGCTGGTTCCTGCCGAAG<br>TTTCCCTCAGGATAGCAAAGACTCGTATCAGTTTTATGAGGTAAAGCGAATGATTAGAGGCCTTGGGGATGAAAC<br>ATCCTTAACCTATTCTCAAACTTTAAATATGTAAGACGCTCCTGTTTCTTAATTGAACGTGGGCATGTGAATGAG<br>AGTCTTTAGTGGGCCATTTTTGGTAAGCAGAACTGGCGATGCGGGATGAACCGAACGTGAGGTTAAGGTGCCGGA<br>ATATACACTCATCAGACACTACAAAAGGTGTTAGTTCATCTAGACAGCCGCACGGTGGCCATGGAAGTCGGAATC<br>CGCTAAGGAGTGTGTAACAACTCAACGGCCGAATGAACTAGCCCTGAAAATGGATGGCGCTTAAGTGTATTACCC<br>ATACCTCACCATTAATATTGTTTTCATTACATATTAATGAGTAGGCAGGCGTGGAGGTTATGTAGCGAAGCCTTG<br>GCAGTGATGCTGGGTGGAACAGCCTCTAGTGCAGATCTTGGTGGAAGTAGCAAATATTCAAGTGAGAACCTTGAA<br>GACTGAAGTGGGGAAGGGTTCCATGGTAACAGCAGTTGGACATGGGTTAG	5.8S-ITS2-28S synthesised sequence for Puccinia striiformis f. sp. Tritici (PST)from GenBank assembly: GCA_001936605.2. Synthesised by GeneWiz.

Nucleic acid name	Sequence	Application
PT_Pt76_28S	TTTCTTAGGACCGACTAACCCATGTCCAACTGCTGTTACCATGGAACCCTTCCCCACTTCAGTCTTCAAGGTTCT<br>CACTTGAATATTTGCTACTTCCACCAAGATCTGCACTAGAGGCTGTTCCACCCAGCATCACTGCCAAGGCTTCGC<br>TACATAACCTCCACGCCTGCCTACTCATTAATATATAAACAACAATATTAATGGTGAGGTATGGGTAATACACTT<br>AAGCGCCATCCATTTTCAGGGCTAGTTCATTCGGCCGTTGAGTTGTTACACACTCCTTAGCGGATTCCGACTTCC<br>ATGGCCACCGTGCGGCTGTCTAGATGAACTAACACCTTTTGTAGTGTCTGATGAGTGTATATTCCGGCACCTTAA<br>CCTCACGTTCGGTTCATCCCGCATCGCCAGTTCTGCTTACCAAAAATGGCCCACTAAAGACTCTCATTCACATGC<br>CCACGTTCAATTAAGAAACAGGAGCGTCTTACATATTTAAAGTTTGAGAATAGGTTAAGGATGTTTCATCCCCAA<br>GGCCTCTAATCATTCGCTTTACCTCATAAAACTGATACGAGTCTTTGCTATCCTGAGGGAAACTTCGGCAGGAAC<br>CAGCTACTAGATGGTTCGATTAGTCTTTCGCCCCTATACCCAAATTTGACGATCGATTTGCACGTCAGAACCGCT<br>ACGAGCTTCCACCAGAGTTTCCTCTGGCTTCACCCTATTCAGGCATAGTTCACCATCTTTCGGGTCCCAACATAC<br>TTGCTCTTACTCAGTACCATCATATAAATAATCTGGACTGGTCAATGGTGCACTTTTGACAGATCCCATTTACAT<br>TTACTTTCATTACGCATAAGGGTTTCAAGCACCCAAATACTTGCAAGCATGTTAGACTCCTTGGTCCGTGTTTCA<br>AGACGGGTCATTTAAAGCTATTACACCAACATCCTCAGTAAGAAGAACATTAATTTGTCCCACACAATAAAGTGC<br>TTACTGCCTTCCTCAATCTTAAGCATTGTATCAAAGCTCAGGACTATAACACAGCCAGAGACTGCTACATTTCCT<br>TAAACCCTTATCCAACACCCAAAAATTGATGTTGGTCTGTTAATCATTAGAATACTCCCTTTAAAAAGGTTGAAC<br>TAATAATAACAAGTCTAACTTCAAGTGTTTCCCTTTTAACAATTTCACGTACTGTTAACTCTCTTTCCAAAGTTC<br>TTTTCATCTTTCCCTCACGGTACTTGTTTGCTATCGGTCTCTCACCTATATTTAGCCTTAGATGGAATTTACCAC<br>CCACTTTGAGCTGCATTCCCAAACAACTCGACTCTTAGAGACTGTATCATAATGCACTGGTAGTCCATATCATGA<br>ACGGGATTATCACCCTCAATGATGCTGCTTTTCAACAGACTTGTACATGGTCCAGCACTGAAAACAGTTCTCAAA<br>ATTACAACTCGGACTCTGAAAGAGCCAGATTACAAATTTGGGCTTTTCCCTCTTCACTCGCCGTTACTAGGGGAA<br>TCCTTGTTAGTTTCTTTTCCTCCGCTTATTGATATGCTTAAGTTCAGCGGGTAGTCCCACCTGATTTGAGGTCTT<br>AAATTTTGAAAAAAGGGGGGTTTTTAGAAGTCCCTTATATTAAACAAGATGGCAAGTATTGCTACTTTCCTTGAT<br>GTGATGATAAAATTATTACACCAAGTCAATCCAACTTATTTGAAAAGTGACTTATATATTTAAAGTGAGCTAATG<br>ACAGCAACACTCAACATCCACTAAACAAGTTTAAACAAGTTAAAAAATGTTTAATGAGAGGGTTTCATGACACTC<br>AAACAGGTGTGCCTTTTGGAATACCAAAAGGCGCAAGATGCGTTCAAAGATTCGATGATTCACTGAATTCTGCAA<br>TTCACATTACTTATCACATTTCACTGTGTTCTTCATCGATGTGAGAGCCTAGAGATCCATTGTTAAAAGTTATTT<br>ATAATTTAAAGGTTGTTTTACATTCATTTAAACTTGTGTTACTTAAAAAA	5.8S-ITS2-28S synthesised sequence for Puccinia triticina (PT)from GenBank assembly: GCA_019358815.1. Synthesised by GeneWiz.
ZT_M3_45_28S	GTCATTTAGAGGAAGTAAAAGTCGTAACAAGGTCTCCGTAGGTGAACCTGCGGAGGGATCATTACCGAGCGAGGG<br>CCTCCGGGTCCGACCTCCAACCCTTTGTGAACACATCCCGTTGCTTCGGGGGCGACCCTGCCGGGCGCCCCCGGA<br>GGACCACCAAAAAACACTGCATCTCTGCGTCGGAGTTTACGAGTAAATCGAAACAAAACTTTCAACAACGGATCT<br>CTTGGTTCTGGCATCGATGAAGAACGCAGCGAAATGCGATAAGTAATGTGAATTGCAGAATTCAGTGAATCATCG<br>AATCTTTGAACGCACATTGCGCCCCCTGGTATTCCGGGGGGCATGCCCGTTCGAGCGTCATTACACCACTCCAGC<br>CTCGCTGGGTATTGGGCGTCTTTTCGCGGGGGATCACTCCCCCGCGCGCCTCAAAGTCTCCGGCTGAGCGGTCTC<br>GTCTCCCAGCGTTGTGGCATCACGTCTCGCCGCGGAGTTCACGAGCCCTCACGGCCGTTAAATCACACCTCAGGT<br>TGACCTCGGATCGGGTAGGGATACCCGCTGAACTTAAGCATATCAATAAGCGGAGGAAAAGAAACCAACAGGGAT<br>TGCCCTAGTAACGGCGAGTGAAGCGGCAACAGCTCAAATTTGAAATCTGGCCCCCCGGCCCGAGTTGTAATTTGT<br>AGAGGATGCTTCTGGGTAGCGACCGGTCTAAGTTCCTTGGAACAGGACGTCATAGAGGGTGAGAATCCCGTATGC<br>GACCGGCCCGCGCCCTCCACGTAGCTCCTTCGACGAGTCGAGTTGTTTGGGAATGCAGCTCTAAATGGGAGGTAA<br>ATTTCTTCTAAAGCTAAATACCGGCCAGAGACCGATAGCGCACAAGTAGAGTGATCGAAAGATGAAAAGCACTTT<br>GGAAAGAGAGTTAAAAAGCACGTGAAATTGTTGAAAGGGAAGCGCTTACAACCAGACTTTGGGGCGGTGTTCCGC<br>CGGTCTTCTGACCGGTCTACTCTCCGTCCCGAGGCCAACATCATCTGGGACCGCCGGACAAGACCTCAGGAATGT<br>AGCTCCCCCTCGGGGGAGTGTTATAGCCTGTGGTGATGCGGCGCGTCCCGGGTGAGGTCCGCGCTTCGGCAAGGA<br>TGTTGGCGTAATGGTTGTCAGCGGCCCGTCTTGAAACACGGACCAAGGAGTCTAACATCTATGCGAGTGTTCGGG<br>TGTCAAACCCCTACGCGGAATGAAAGTGAACGGAGGTGGGAAGGGGCAACCCTGCACCATCGACCGATCCTGATG<br>TCCTCGGATGGATTTGAGTAAGAGCATAGCTGTTGGGACCCGAAAGATGGTGAACTATGCCTGAATAGGGTGAAG<br>CCAGAGGAAACTCTGGTGGAGGCTCGCAGCGGTTCTGACGTGCAAATCGATCGTCAAATTTGGGTATAGGGGCGA<br>AAGACTAATCGAACCATCTAGTAGCTGGTTCCTGCCGAAGTTTCCCTCAGGATAGCAGTAACGTTTTCAGTTTTA<br>TGAGGTAAAGCGAATGATTAGAGGCCTTGGGGTTGAAACAACCTTAACCTATTCTCAAACTTTAAATATGTAAGA<br>AGTCCTTGTTACTTAGTTGAACGTGGACATTTGAATGTATCGTTACTAGTGGGCCATTTTTGGTAAGCAGAACTG<br>GCGATGCGGGATGAACCGAACGCGAGGTTAAGGTGCCGGAATGTACGCTCATCAGACACCACAAAAGGTGTTAGT<br>TCATCTAGACAGCAGGACGGTGGCCATGGAAGTCGGAATCCGCTAAGGAGTGTGTAACAACTCACCTGCCGAATG<br>AACTAGCCCTGAAAATGGATGGCGCTTAAGCGTGCTACCCATACCTCGCCGCCAGGGTAGAAACGATGCCCTGGC<br>GAGTAGGCAGGCGTGGAGGTCCGTGACGAAGCCTTGGGGGTGACCCCGGGTCGAACGGCCTCTAGTGCAGATCTT<br>GGTGGTAGTAGCAAATACTCAAATGAGAACTTTGAGGACTGAAGTGGGGAAAGGTTCCGT	5.8S-ITS2-28S synthesised sequence for Zymoseptoria tritici(ZT)from GenBank assembly: GCA_017766645.1. Synthesised by GeneWiz.
pUC-GW-AMP	TCGCGCGTTTCGGTGATGACGGTGAAAACCTCTGACACATGCAGCTCCCGGAGACTGTCACAGCTTGTCTGTAAG<br>CGGATGCCGGGAGCAGACAAGCCCGTCAGGGCGCGTCAGCGGGTGTTGGCGGGTGTCGGGGCTGGCTTAACTATG<br>CGGCATCAGAGCAGATTGTACTGAGAGTGCACCATATGCGGTGTGAAATACCGCACAGATGCGTAAGGAGAAAAT<br>ACCGCATCAGGCGCCATTCGCCATTCAGGCTGCGCAACTGTTGGGAAGGGCGATCGGTGCGGGCCTCTTCGCTAT<br>TACGCCAGCTGGCGAAAGGGGGATGTGCTGCAAGGCGATTAAGTTGGGTAACGCCAGGGTTTTCCCAGTCACGAC<br>GTTGTAAAACGACGGCCAGTGAATTGACGCGTATTGGGATATCCCAATGGCGCGCCGAGCTTGGCGTAATCATGG<br>TCATAGCTGTTTCCTGTGTGAAATTGTTATCCGCTCACAATTCCACACAACATACGAGCCGGAAGCATAAAGTGT<br>AAAGCCTGGGGTGCCTAATGAGTGAGCTAACTCACATTAATTGCGTTGCGCTCACTGCCCGCTTTCCAGTCGGGA<br>AACCTGTCGTGCCAGCTGCATTAATGAATCGGCCAACGCGCGGGGAGAGGCGGTTTGCGTATTGGGCGCTGTTCC<br>GCTTCCTCGCTCACTGACTCGCTGCGCTCGGTCGTTCGGCTGCGGCGAGCGGTATCAGCTCACTCAAAGGCGGTA<br>ATACGGTTATCCACAGAATCAGGGGATAACGCAGGAAAGAACATGTGAGCAAAAGGCCAGCAAAAGGCCAGGAAC<br>CGTAAAAAGGCCGCGTTGCTGGCGTTTTTCCATAGGCTCCGCCCCCCTGACGAGCATCACAAAAATCGACGCTCA<br>AGTCAGAGGTGGCGAAACCCGACAGGACTATAAAGATACCAGGCGTTTCCCCCTGGAAGCTCCCTCGTGCGCTCT<br>CCTGTTCCGACCCTGCCGCTTACCGGATACCTGTCCGCCTTTCTCCCTTCGGGAAGCGTGGCGCTTTCTCATAGC<br>TCACGCTGTAGGTATCTCAGTTCGGTGTAGGTCGTTCGCTCCAAGCTGGGCTGTGTGCACGAACCCCCCGTTCAG<br>CCCGACCGCTGCGCCTTATCCGGTAACTATCGTCTTGAGTCCAACCCGGTAAGACACGACTTATCGCCACTGGCA<br>GCAGCCACTGGTAACAGGATTAGCAGAGCGAGGTATGTAGGCGGTGCTACAGAGTTCTTGAAGTGGTGGCCTAAC<br>TACGGCTACACTAGAAGAACAGTATTTGGTATCTGCGCTCTGCTGAAGCCAGTTACCTTCGGAAAAAGAGTTGGT<br>AGCTCTTGATCCGGCAAACAAACCACCGCTGGTAGCGGTGGTTTTTTTGTTTGCAAGCAGCAGATTACGCGCAGA<br>AAAAAAGGATCTCAAGAAGATCCTTTGATCTTTTCTACGGGGTCTGACGCTCAGTGGAACGAAAACTCACGTTAA<br>GGGATTTTGGTCATGAGATTATCAAAAAGGATCTTCACCTAGATCCTTTTAAATTAAAAATGAAGTTTTAAATCA<br>ATCTAAAGTATATATGAGTAAACTTGGTCTGACAGTTACCAATGCTTAATCAGTGAGGCACCTATCTCAGCGATC<br>TGTCTATTTCGTTCATCCATAGTTGCCTGACTCCCCGTCGTGTAGATAACTACGATACGGGAGGGCTTACCATCT<br>GGCCCCAGTGCTGCAATGATACCGCGAGAACCACGCTCACCGGCTCCAGATTTATCAGCAATAAACCAGCCAGCC<br>GGAAGGGCCGAGCGCAGAAGTGGTCCTGCAACTTTATCCGCCTCCATCCAGTCTATTAATTGTTGCCGGGAAGCT<br>AGAGTAAGTAGTTCGCCAGTTAATAGTTTGCGCAACGTTGTTGCCATTGCTACAGGCATCGTGGTGTCACGCTCG<br>TCGTTTGGTATGGCTTCATTCAGCTCCGGTTCCCAACGATCAAGGCGAGTTACATGATCCCCCATGTTGTGCAAA<br>AAAGCGGTTAGCTCCTTCGGTCCTCCGATCGTTGTCAGAAGTAAGTTGGCCGCAGTGTTATCACTCATGGTTATG<br>GCAGCACTGCATAATTCTCTTACTGTCATGCCATCCGTAAGATGCTTTTCTGTGACTGGTGAGTACTCAACCAAG<br>TCATTCTGAGAATAGTGTATGCGGCGACCGAGTTGCTCTTGCCCGGCGTCAATACGGGATAATACCGCGCCACAT<br>AGCAGAACTTTAAAAGTGCTCATCATTGGAAAACGTTCTTCGGGGCGAAAACTCTCAAGGATCTTACCGCTGTTG<br>AGATCCAGTTCGATGTAACCCACTCGTGCACCCAACTGATCTTCAGCATCTTTTACTTTCACCAGCGTTTCTGGG<br>TGAGCAAAAACAGGAAGGCAAAATGCCGCAAAAAAGGGAATAAGGGCGACACGGAAATGTTGAATACTCATACTC<br>TTCCTTTTTCAATATTATTGAAGCATTTATCAGGGTTATTGTCTCATGAGCGGATACATATTTGAATGTATTTAG<br>AAAAATAAACAAATAGGGGTTCCGCGCACATTTCCCCGAAAAGTGCCACCTGACGTCTAAGAAACCATTATTATC<br>ATGACATTAACCTATAAAAATAGGCGTATCACGAGGCCCTTTTGTC	Plasmid vector used for cloning 5.8S-ITS2-28S target sequence. Addgene plasmid # 164506.

S1 Table 2. Concentration of each guide RNA transcription reaction following purification. Quantification was performed using Qubit RNA Broad Range kit.

gRNA	Transcription A (ng/ul)	Transcription B (ng/ul)	Method deployment
**EnGen sgRNAs** 1	642.8	561.6	EnGEN sgRNAs
**EnGen sgRNAs** 2	915.8	731.8	
**EnGen sgRNAs** 3	574.5	403.8	
**EnGen sgRNAs** 4	1091	1194.4	
**EnGen sgRNAs** 5	685.2	719	
**EnGen sgRNAs** 6	1090	1153	
**EnGen sgRNAs** 7	159	256.8	
**EnGen sgRNAs** 8	334.8	241.8	
**Pooled EnGEN sgRNAs**	905.9	812.3	Pooled EnGen sgRNAs
**HiScribe crRNA 1**	129	193.6	HiScribe cr:tracrRNAs
**HiScribe crRNA 2**	3153	3172	
**HiScribe crRNA 3**	216.2	526	
**HiScribe crRNA 4**	2860	2946	
**HiScribe crRNA 5**	195.6	336.1	
**HiScribe crRNA 6**	2174	2217	
**HiScribe crRNA 7**	218.5	554.7	
**HiScribe crRNA 8**	99.42	123.2	
**HiScribe pooled crRNAs**	2516	2358	Pooled HiScribe cr:tracrRNA
**HiScribe tracrRNA**	2392	2386	HiScribe cr:tracrRNAs and Pooled HiScribe cr:tracrRNA

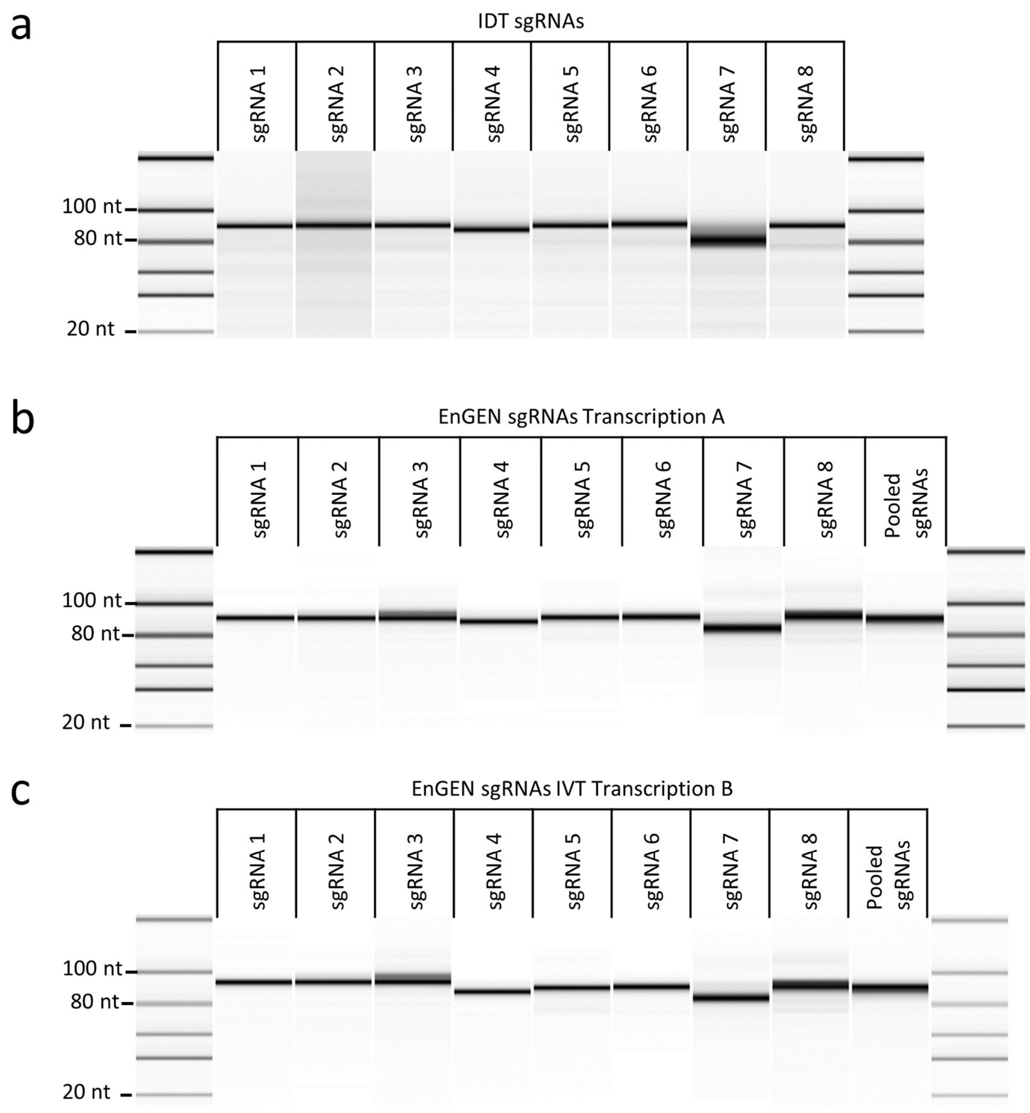

S1 Fig 1. Evaluation of sgRNA integrity using the Agilent Bioanalyzer Small RNA Kit. The simulated gel images derived from electropherograms shows the integrity of the sgRNAs, including: (a) Commercially synthesised sgRNAs from IDT; (b) NEB EnGEN-produced individual and pooled sgRNAs from transcription set A; and (c) EnGEN-produced individual and pooled sgRNAs from transcription set B.

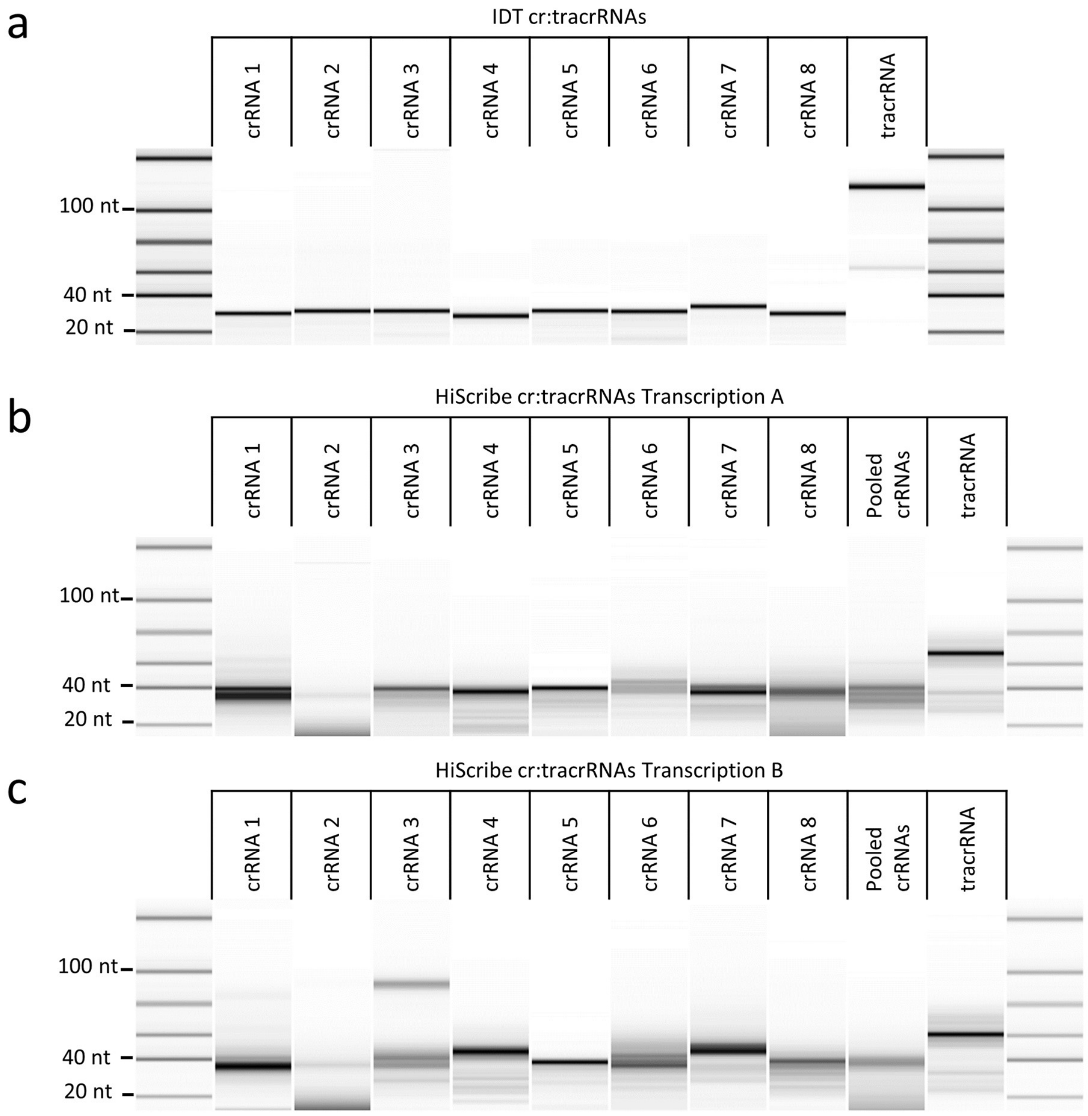

S1 Fig 2. Evaluation of crRNA and tracrRNA integrity using the Agilent Bioanalyzer Small RNA Kit. The simulated gel images derived from electropherograms shows the integrity of the crRNAs and tracrRNAs, including: (a) Commercially synthesised crRNAs and tracrRNA from IDT. The unanticipated increase in the molecular weight of IDT's tracrRNA is likely due to proprietary chemical stabilisation modifications that IDT integrates into their tracrRNA molecules.; (b) HiScribe-generated individual and pooled crRNAs and tracrRNA from transcription set A; (c) HiScribe-generated individual and pooled crRNAs and tracrRNA from transcription set B. The additional band observed in crRNA 3 at approximately 100 nt could be attributed to either template contamination or the formation of double-stranded RNA (dsRNA).

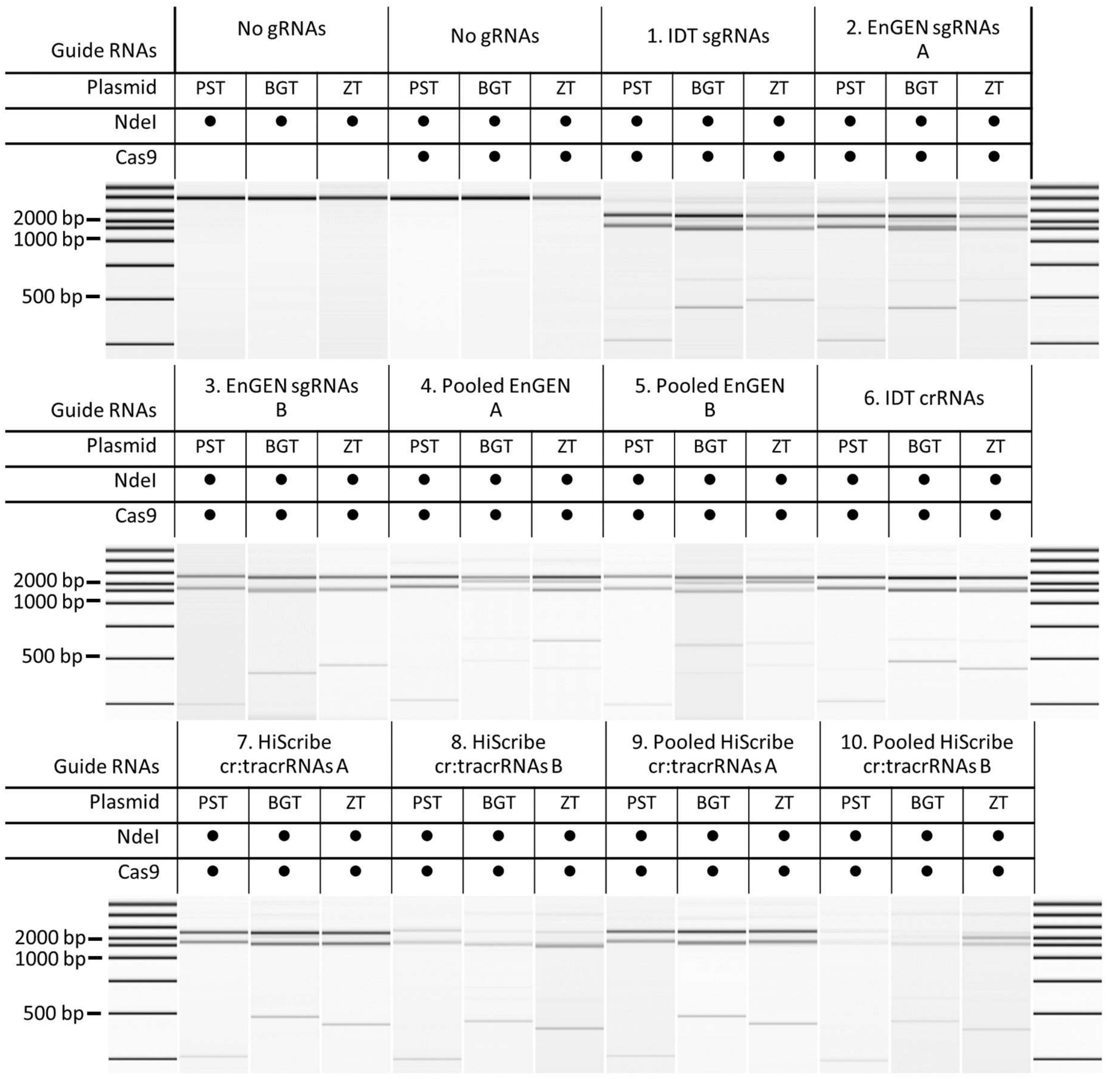

S1 Fig 3. Assessing each gRNA library's ability to cleave PST, BGT, and ZT plasmids. Collectively, these three plasmids contain all eight gRNA recognition sites. These simulated gel images were generated from electropherograms using Agilent Bioanalyzer DNA 7500 kit. Plasmids were digested with NdeI, Cas9, and one of the ten gRNA libraries. Controls included digestion with NdeI only (without Cas9 or gRNAs) and NdeI with Cas9 (without gRNAs). All libraries demonstrated the expected banding pattern, albeit with varying band intensities.

S1 Table 3a. Comparative summary of costs for producing 1 nmol of an individual guide RNA using four different guide RNA production methods.

Guide RNA production method	Approximate cost of 1 nmol of a single guide RNA
IDT sgRNA	AU$135
IDT cr:tracr	AU$84
EnGEN sgRNA	AU$52
HiScribe cr:tracrRNA	AU$29

S1 Table 3b. Cost calculations for purchasing 1 nmol of IDT sgRNA.

Items	Unit size	Cost	Cost per 1 nmol
IDT sgRNA	2 nmol	AU$269.00	AU$134.50
		**Total**	AU$134.50

S1 Table 3c. Cost calculations for purchasing 1 nmol of IDT cr:tracrRNA.

Items	Unit size	Cost	Cost per 1 nmol
IDT crRNA	2 nmol	AU$119.00	AU$59.50
IDT tracrRNA	5 nmol	AU$122.00	AU$24.40
		**Total**	AU$83.90

S1 Table 3d. Cost calculations for producing 1 nmol of EnGen sgRNA.

Items for sgRNA transcription	Unit size	Cost	Cost per reaction
IDT DNA oligo 55 nt	25 nmol	AU$37.00	AU$37.00
NEB EnGen sgRNA kit	20 reactions	AU$894.00	AU$44.70
NEB Monarch RNA Cleanup (50 µg) Kit	100 reactions	AU$506.85	AU$5.07
		**Total**	AU$86.77
	**Mean yield per transcription reaction**		1.661 nmol
		**Cost per 1 nmol**	AU$52.24

S1 Table 3e. Cost calculation for producing 1 nmol of HiScribe cr:tracrRNA.

Items for crRNA transcription	Unit size	Cost	Cost per reaction
IDT DNA oligo 60 nt	25 nmol	AU$40.00	AU$40.00
NEB HiScribe T7 Quick High Yield RNA Synthesis Kit	250 reactions	AU$2,333.00	AU$9.33
NEB Monarch RNA Cleanup (50 µg) Kit	100 reactions	AU$506.85	AU$5.07
		**Total**	AU$54.40
		**Mean yield per transcription reaction**	5.2 nmol
		**Cost per 1 nmol**	AU$10.46
**Items for tracrRNA transcription**	**Unit size**	**Cost**	**Cost per reaction**
IDT DNA oligo 90nt	100 nmol	AU$83.70	AU$83.70
NEB HiScribe T7 Quick High Yield RNA Synthesis Kit	250 reactions	AU$2,333.00	AU$9.33
NEB Monarch RNA Cleanup (50 µg) Kit	100 reactions	AU$506.85	AU$5.07
		**Total**	AU$98.10
		**Mean yield per transcription reaction**	5.2 nmol
		**Cost per 1 nmol**	AU$18.87
		**Total cost of 1 nmol of cr:tracrRNA duplex**	AU$29.33

S1 Table 4. Alignment of BGT_HiScribe_cr:tracrRNA consensus sequences from run 1 and 2 to the PST_5.8S-ITS2-28S_reference sequence

```
                                       10        20        30        40        50        60        70        80        90       100       110       120       130       140       150
BGT_5.8S-ITS2-28S_Reference    GTTCGAGCGTCCGTAACAACCTCTCAAGCCTGGCTTGGTATTGGGACTCGCTGCCTCGGTGGTGGTCCTCAAAAGCAGTGGCGGGCCCATGTAACTCTCCGCGTAGTAATACATCTCGCGACAGAGAAGCAGCGGGACTTGCCAAAACTC
BGT_HiScribe_cr:tracrRNA_Run_2 ......................................................................................................................................................
BGT_HiScribe_cr:tracrRNA_Run_1 ......................................................................................................................................................

                                      160       170       180       190       200       210       220       230       240       250       260       270       280       290       300
BGT_5.8S-ITS2-28S_Reference    CTTAATTGCTCAGGTTGACCTCGAATCAGGTAGGGATACCCGCTGAACTTAAGCATATCAATAAGCGGAGGAAAAGAAACCAACAGGGATTACCTCAGTAACGGCGAGTGAAGCGGTAACAGCTCAAATTTGAAATCTGGTCCGTTTACG
BGT_HiScribe_cr:tracrRNA_Run_2 ......................................................................................................................................................
BGT_HiScribe_cr:tracrRNA_Run_1 ......................................................................................................................................................

                                      310       320       330       340       350       360       370       380       390       400       410       420       430       440       450
BGT_5.8S-ITS2-28S_Reference    GGCCCGAGTTGTAATTTGTAGAAGATGCTTTGGTGACGAGTCCGGCCTAAGTTCCTTGGAACAGGACGTCATAGAGGGTGAGAACCCGTATGCGGCCGGGTGCTCGATGCTATGTAAAGCTCTTTCGACGAGTCGAGTTGTTTGGGAATG
BGT_HiScribe_cr:tracrRNA_Run_2 ......................................................................................................................................................
BGT_HiScribe_cr:tracrRNA_Run_1 ......................................................................................................................................................

                                      460       470       480       490       500       510       520       530       540       550       560       570       580       590       600
BGT_5.8S-ITS2-28S_Reference    CAGCTCAAAATGGGTGGTAAATTTCATCTAAAGCTAAATATTGGCCAGAGACCGATAGCGCACAAGTAGAGTGATCGAAAGATGAAAAGCACTTTGGAAAGAGAGTTAAACAGTACGTGAAATTGTTGAAAGGGAAGCGCTTGCAACCAG
BGT_HiScribe_cr:tracrRNA_Run_2 ......................................................................................................................................................
BGT_HiScribe_cr:tracrRNA_Run_1 ......................................................................................................................................................

                                      610       620       630       640       650       660       670       680       690       700       710       720       730       740       750
BGT_5.8S-ITS2-28S_Reference    ACTTGGGCACTGTGGATCATCCGAGGTTCTCCTCGGTGCACTCGGCAGTGCGCAGGCCAGCATCAGTTTGGGTGGTTGGATAAAGACTTGTGGAATGTAGCTCCTTTCGGGAGTGTTATAGCCACAGGTGCCATGCGACCAACCCGGACT
BGT_HiScribe_cr:tracrRNA_Run_2 ......................................................................................................................................................
BGT_HiScribe_cr:tracrRNA_Run_1 ......................................................................................................................................................

                                      760       770       780       790       800       810       820       830       840       850       860       870       880       890       900
BGT_5.8S-ITS2-28S_Reference    GAGGACCGCGCTTCGGCTAGGATGCTGGCGTAATGGTTGTAAGCGACCCGTCTTGAAACACGGACCAAGGAGTCTAACATCTATGCGAGTGTTTGGGTGTTAAACCCATACGCGGAATGAAAGTGAACGTAGGTGAGAACCCATAAGGGG
BGT_HiScribe_cr:tracrRNA_Run_2 ......................................................................................................................................................
BGT_HiScribe_cr:tracrRNA_Run_1 ......................................................................................................................................................

                                      910       920       930       940       950       960       970       980       990      1000      1010      1020      1030      1040      1050
BGT_5.8S-ITS2-28S_Reference    GCATCATCGACCGATCCGGATGTCTTCGGATGGATTTGAGTAAGAGCATAGCTGTTGGGACCCGAAAGATGGTGAACTATGCCTGAATAGGGTGAAGCCAGAGGAAACTCTGGTGGAGGCTCGCAGCGGTTCTGACGTGCAAATCGATCG
BGT_HiScribe_cr:tracrRNA_Run_2 ......................................................................................................................................................
BGT_HiScribe_cr:tracrRNA_Run_1 ......................................................................................................................................................

                                     1060      1070      1080      1090      1100      1110      1120      1130      1140      1150      1160      1170      1180      1190      1200
BGT_5.8S-ITS2-28S_Reference    TCAAATTTGGGTATAGGGGCGAAAGACTAATCGAACCATCTAGTAGCTGGTTCCTGCCGAAGTTTCCCTCAGGATAGCAGTGTTGAATTCAGTTTTATGAGGTAAAGCGAATGATTAGAGGCCTTGGGGTTGAAACAACCTTAACCTATT
BGT_HiScribe_cr:tracrRNA_Run_2 ......................................................................................................................................................
BGT_HiScribe_cr:tracrRNA_Run_1 ......................................................................................................................................................

                                     1210      1220      1230      1240      1250      1260      1270      1280      1290      1300      1310      1320      1330      1340      1350
BGT_5.8S-ITS2-28S_Reference    CTCAAACTTTAAATATGTAAGAAGTCCTTGTTACTTAATTGAACGTGGACAGCCGAATGTACCAACACTAGTGGGCCATTTTTGGTAAGCAGAACTGGCGATGCGGGATGAACCGAACGTGAAGTTAAGGTGCCGGAATACACGCTCATC
BGT_HiScribe_cr:tracrRNA_Run_2 ......................................................................................................................................................
BGT_HiScribe_cr:tracrRNA_Run_1 ......................................................................................................................................................

                                     1360      1370      1380      1390      1400      1410      1420      1430      1440      1450      1460      1470      1480      1490      1500
BGT_5.8S-ITS2-28S_Reference    AGACACCACAAAAGGTGTTAGTTCATCTAGACAGCAGGACGGTGGCCATGGAAGTCGGAATCCGCTAAGGAGTGTGTAACAACTCACCTGCCGAATGAACTAGCCCTGAAAATGGATGGCGCTTAAGCGTGTTACCCATACTTCACCGCC
BGT_HiScribe_cr:tracrRNA_Run_2 ......................................................................................................................................................
BGT_HiScribe_cr:tracrRNA_Run_1 ......................................................................................................................................................

                                     1510      1520      1530      1540      1550      1560      1570      1580
BGT_5.8S-ITS2-28S_Reference    CGGGTAGAAACGATGCCCGGGCGAGTAGGCAGGCGTGGAGGTCAGTGACGAAGCCTTGGGAGTGATCCCGGGTCGAACGGCCTCTA
BGT_HiScribe_cr:tracrRNA_Run_2 ......................................................................................
BGT_HiScribe_cr:tracrRNA_Run_1 ......................................................................................
```

S1 Table 5. Alignment of PT_HiScribe_cr:tracrRNA consensus sequences from run 1 and 2 to the PST_5.8S-ITS2-28S_reference sequence

```
                                      10        20        30        40        50        60        70        80        90       100       110       120       130       140       150
PT_5.8S-ITS2-28S_Reference    TAGAGGCTGTTCCACCCAGCATCACTGCCAAGGCTTCGCTACATAACCTCCACGCCTGCCTACTCATTAATATATAAACAACAATATTAATGGTGAGGTATGGGTAATACACTTAAGCGCCATCCATTTTCAGGGCTAGTTCATTCGGCC
PT_HiScribe_cr:tracrRNA_Run_2 ......................................................................................................................................................
PT_HiScribe_cr:tracrRNA_Run_1 ......................................................................................................................................................

                                     160       170       180       190       200       210       220       230       240       250       260       270       280       290       300
PT_5.8S-ITS2-28S_Reference    GTTGAGTTGTTACACACTCCTTAGCGGATTCCGACTTCCATGGCCACCGTGCGGCTGTCTAGATGAACTAACACCTTTTGTAGTGTCTGATGAGTGTATATTCCGGCACCTTAACCTCACGTTCGGTTCATCCCGCATCGCCAGTTCTGC
PT_HiScribe_cr:tracrRNA_Run_2 ......................................................................................................................................................
PT_HiScribe_cr:tracrRNA_Run_1 ......................................................................................................................................................

                                     310       320       330       340       350       360       370       380       390       400       410       420       430       440       450
PT_5.8S-ITS2-28S_Reference    TTACCAAAAATGGCCCACTAAAGACTCTCATTCACATGCCCACGTTCAATTAAGAAACAGGAGCGTCTTACATATTTAAAGTTTGAGAATAGGTTAAGGATGTTTCATCCCCAAGGCCTCTAATCATTCGCTTTACCTCATAAAACTGAT
PT_HiScribe_cr:tracrRNA_Run_2 ......................................................................................................................................................
PT_HiScribe_cr:tracrRNA_Run_1 ......................................................................................................................................................

                                     460       470       480       490       500       510       520       530       540       550       560       570       580       590       600
PT_5.8S-ITS2-28S_Reference    ACGAGTCTTTGCTATCCTGAGGGAAACTTCGGCAGGAACCAGCTACTAGATGGTTCGATTAGTCTTTCGCCCCTATACCCAAATTTGACGATCGATTTGCACGTCAGAACCGCTACGAGCTTCCACCAGAGTTTCCTCTGGCTTCACCCT
PT_HiScribe_cr:tracrRNA_Run_2 ......................................................................................................................................................
PT_HiScribe_cr:tracrRNA_Run_1 ......................................................................................................................................................

                                     610       620       630       640       650       660       670       680       690       700       710       720       730       740       750
PT_5.8S-ITS2-28S_Reference    ATTCAGGCATAGTTCACCATCTTTCGGGTCCCAACATACTTGCTCTTACTCAGTACCATCATATAAATAATCTGGACTGGTCAATGGTGCACTTTTGACAGATCCCATTTACATTTACTTTCATTACGCATAAGGGTTTCAAGCACCCAA
PT_HiScribe_cr:tracrRNA_Run_2 ......................................................................................................................................................
PT_HiScribe_cr:tracrRNA_Run_1 ......................................................................................................................................................

                                     760       770       780       790       800       810       820       830       840       850       860       870       880       890       900
PT_5.8S-ITS2-28S_Reference    ATACTTGCAAGCATGTTAGACTCCTTGGTCCGTGTTTCAAGACGGGTCATTTAAAGCTATTACACCAACATCCTCAGTAAGAAGAACATTAATTTGTCCCACACAATAAAGTGCTTACTGCCTTCCTCAATCTTAAGCATTGTATCAAAG
PT_HiScribe_cr:tracrRNA_Run_2 ......................................................................................................................................................
PT_HiScribe_cr:tracrRNA_Run_1 ......................................................................................................................................................

                                     910       920       930       940       950       960       970       980       990      1000      1010      1020      1030      1040      1050
PT_5.8S-ITS2-28S_Reference    CTCAGGACTATAACACAGCCAGAGACTGCTACATTTCCTTAAACCCTTATCCAACACCCAAAAATTGATGTTGGTCTGTTAATCATTAGAATACTCCCTTTAAAAAGGTTGAACTAATAATAACAAGTCTAACTTCAAGTGTTTCCCTTT
PT_HiScribe_cr:tracrRNA_Run_2 ......................................................................................................................................................
PT_HiScribe_cr:tracrRNA_Run_1 ......................................................................................................................................................

                                    1060      1070      1080      1090      1100      1110      1120      1130      1140      1150      1160      1170      1180      1190      1200
PT_5.8S-ITS2-28S_Reference    TAACAATTTCACGTACTGTTAACTCTCTTTCCAAAGTTCTTTTCATCTTTCCCTCACGGTACTTGTTTGCTATCGGTCTCTCACCTATATTTAGCCTTAGATGGAATTTACCACCCACTTTGAGCTGCATTCCCAAACAACTCGACTCTT
PT_HiScribe_cr:tracrRNA_Run_2 ......................................................................................................................................................
PT_HiScribe_cr:tracrRNA_Run_1 ......................................................................................................................................................

                                    1210      1220      1230      1240      1250      1260      1270      1280      1290      1300      1310      1320      1330      1340      1350
PT_5.8S-ITS2-28S_Reference    AGAGACTGTATCATAATGCACTGGTAGTCCATATCATGAACGGGATTATCACCCTCAATGATGCTGCTTTTCAACAGACTTGTACATGGTCCAGCACTGAAAACAGTTCTCAAAATTACAACTCGGACTCTGAAAGAGCCAGATTACAAA
PT_HiScribe_cr:tracrRNA_Run_2 ......................................................................................................................................................
PT_HiScribe_cr:tracrRNA_Run_1 ......................................................................................................................................................

                                    1360      1370      1380      1390      1400      1410      1420      1430      1440      1450      1460      1470      1480      1490      1500
PT_5.8S-ITS2-28S_Reference    TTTGGGCTTTTCCCTCTTCACTCGCCGTTACTAGGGGAATCCTTGTTAGTTTCTTTTCCTCCGCTTATTGATATGCTTAAGTTCAGCGGGTAGTCCCACCTGATTTGAGGTCTTAAATTTTGAAAAAAGGGGGTTTTTAGAAGTCCCTT
PT_HiScribe_cr:tracrRNA_Run_2 .................................N..............................................................................................N.....................
PT_HiScribe_cr:tracrRNA_Run_1 .................................N..............................................................................................N.....................

                                    1510      1520      1530      1540      1550      1560      1570      1580      1590      1600      1610      1620      1630      1640      1650
PT_5.8S-ITS2-28S_Reference    ATATTAAACAAGATGGCAAGTATTGCTACTTTCCTTGATGTGATGATAAAATTATTACACCAAGTCAATCCAACTTATTTGAAAAGTGACTTATATATTTAAAGTGAGCTAATGACAGCAACACTCAACATCCACTAAACAAGTTTAAAC
PT_HiScribe_cr:tracrRNA_Run_2 ......................................................................................................................................................
PT_HiScribe_cr:tracrRNA_Run_1 ......................................................................................................................................................

                                    1660      1670      1680      1690
PT_5.8S-ITS2-28S_Reference    AAGTTAAAAAATGTTTAATGAGAGGGTTTCATGACACTCA
PT_HiScribe_cr:tracrRNA_Run_2 ........................................
PT_HiScribe_cr:tracrRNA_Run_1 ........................................
```

S1 Table 6. Alignment of PST_HiScribe_cr:tracrRNA consensus sequences from run 1 and 2 to the PST_5.8S-ITS2-28S_reference sequence

```
                                       10        20        30        40        50        60        70        80        90       100       110       120       130       140       150
PST_5.8S-ITS2-28S_Reference    TGAGTGTCATGAAACCCTCTCATTAAATAATTTTGATTAATTATTTCAATGGATGTTGAGTGCTGCTGTAATTAGCTCACTTTAAATATATAAGTCACTTTTCTATAAGTTGGATTGACTTGGTGTAATAATTTTATCATCACATCAAG
PST_HiScribe_cr:tracrRNA_Run_2 ......................................................................................................................................................
PST_HiScribe_cr:tracrRNA_Run_1 ......................................................................................................................................................

                                      160       170       180       190       200       210       220       230       240       250       260       270       280       290       300
PST_5.8S-ITS2-28S_Reference    GATTGTAGCAATACTGCCATCTTATTTAAGGGAGACTCCTAAAAACCCAATTTTAACCTTAAGACCTCAAATCAGGTGGGACTACCCGCTGAACTTAAGCATATCAATAAGCGGAGGAAAAGAAACTAACAAGGATTCCCCTAGTAACGG
PST_HiScribe_cr:tracrRNA_Run_2 .........................................................................................................................................N............
PST_HiScribe_cr:tracrRNA_Run_1 .............................N........................................................................................................................

                                      310       320       330       340       350       360       370       380       390       400       410       420       430       440       450
PST_5.8S-ITS2-28S_Reference    CGAGTGAAGAGGGAAAAGCCCAAATTTGTAATCTGGCTCTTTCAGAGTCCGAGTTGTAATTTTGAGAACTGTTTTCAGTGCTGGACCATGTATAAGTCTGTTGAAAAGCAGCATCATTGAGGGTGATAATCCCGTTCATGATATGGACTA
PST_HiScribe_cr:tracrRNA_Run_2 ......................................................................................................................................................
PST_HiScribe_cr:tracrRNA_Run_1 ......................................................................................................................................................

                                      460       470       480       490       500       510       520       530       540       550       560       570       580       590       600
PST_5.8S-ITS2-28S_Reference    CCAGTGCATTATGATACAGTCTCTAAGAGTCGAGTTGTTTGGGAATGCAGCTCAAAGTGGGTGGTAAATTCCATCTAAGGCTAAATATAGGTGAGAGACCGATAGCAAACAAGTACCGTGAGGGAAAGATGAAAAGAACTTTGGAAAGAG
PST_HiScribe_cr:tracrRNA_Run_2 ......................................................................................................................................................
PST_HiScribe_cr:tracrRNA_Run_1 ......................................................................................................................................................

                                      610       620       630       640       650       660       670       680       690       700       710       720       730       740       750
PST_5.8S-ITS2-28S_Reference    AGTTAACAGTACGTGAAATTGTTAAAAGGGAAACACTTGAAGTTAGACTTGTTATTATTAGTTCAACCTTTTTGACGAGGGAGTATTCTAATGATTAACAGACCAACATCAATTTTTGGGTGTTGGAGAAGGGTTTAAGGAAATGTAGCA
PST_HiScribe_cr:tracrRNA_Run_2 ......................................................................................................................................................
PST_HiScribe_cr:tracrRNA_Run_1 ......................................................................................................................................................

                                      760       770       780       790       800       810       820       830       840       850       860       870       880       890       900
PST_5.8S-ITS2-28S_Reference    GTCTCTGACTGTGTTATAGTCCTGAGCTTTGATACAATGCTTAAGATTGAGGAAGGCAGTAAGCGCAATTTATTGTGTGGAACAAATTAATGTTCTTCTTACTGAGGATGTTGGTGTAATAGCTTTAAATGACCCGTCTTGAAACACGGA
PST_HiScribe_cr:tracrRNA_Run_2 ......................................................................................................................................................
PST_HiScribe_cr:tracrRNA_Run_1 ......................................................................................................................................................

                                      910       920       930       940       950       960       970       980       990      1000      1010      1020      1030      1040      1050
PST_5.8S-ITS2-28S_Reference    CCAAGGAGTCTAACATGCTTGCAAGTATTTGGGTGCTTGAAACCCTTATGCGTAATGAAAGTAAATGTAAATGGGATCTGTTAAAAGTGCACCATTGACCAGTCCAGATTATTTATATGATGGTACTGAGTAAGAGCAAGTATGTTGGGA
PST_HiScribe_cr:tracrRNA_Run_2 ......................................................................................................................................................
PST_HiScribe_cr:tracrRNA_Run_1 ......................................................................................................................................................

                                     1060      1070      1080      1090      1100      1110      1120      1130      1140      1150      1160      1170      1180      1190      1200
PST_5.8S-ITS2-28S_Reference    CCCGAAAGATGGTGAACTATGCCTGAATAGGGTGAAGCCAGAGGAAACTCTGGTGGAAGCTCGTAGCGGTTCTGACGTGCAAATCGATCGTCAAATTTGGGTATAGGGGCGAAAGACTAATCGAACCATCTAGTAGCTGGTTCCTGCCGA
PST_HiScribe_cr:tracrRNA_Run_2 ......................................................................................................................................................
PST_HiScribe_cr:tracrRNA_Run_1 ......................................................................................................................................................

                                     1210      1220      1230      1240      1250      1260      1270      1280      1290      1300      1310      1320      1330      1340      1350
PST_5.8S-ITS2-28S_Reference    AGTTTCCCTCAGGATAGCAAAGACTCGTATCAGTTTTATGAGGTAAAGCGAATGATTAGAGGCCTTGGGGATGAAACATCCTTAACCTATTCTCAAACTTTAAATATGTAAGACGCTCCTGTTTCTTAATTGAACGTGGGCATGTGAATG
PST_HiScribe_cr:tracrRNA_Run_2 ......................................................................................................................................................
PST_HiScribe_cr:tracrRNA_Run_1 ......................................................................................................................................................

                                     1360      1370      1380      1390      1400      1410      1420      1430      1440      1450      1460      1470      1480      1490      1500
PST_5.8S-ITS2-28S_Reference    AGAGTCTTTAGTGGGCCATTTTTGGTAAGCAGAACTGGCGATGCGGGATGAACCGAACGTGAGGTTAAGGTGCCGGAATATACACTCATCAGACACTACAAAAGGTGTTAGTTCATCTAGACAGCCGCACGGTGGCCATGGAAGTCGGAA
PST_HiScribe_cr:tracrRNA_Run_2 ......................................................................................................................................................
PST_HiScribe_cr:tracrRNA_Run_1 ......................................................................................................................................................

                                     1510      1520      1530      1540      1550      1560      1570      1580      1590      1600      1610      1620      1630      1640      1650
PST_5.8S-ITS2-28S_Reference    TCCGCTAAGGAGTGTGTAACAACTCAACGGCCGAATGAACTAGCCCTGAAAATGGATGGCGCTTAAGTGTATTACCCATACCTCACCATTAATATTGTTTTCATTACATATTAATGAGTAGGCAGGCGTGGAGGTTATGTAGCGAAGCCT
PST_HiScribe_cr:tracrRNA_Run_2 ......................................................................................................................................................
PST_HiScribe_cr:tracrRNA_Run_1 ......................................................................................................................................................

                                     1660      1670      1680
PST_5.8S-ITS2-28S_Reference    TGGCAGTGATGCTGGGTGGAACAGCCTCTA
PST_HiScribe_cr:tracrRNA_Run_2 ..............................
PST_HiScribe_cr:tracrRNA_Run_1 ..............................
```

S1 Table 7. Alignment of ZT_HiScribe_cr:tracrRNA consensus sequences from run 1 and 2 to the PST_5.8S-ITS2-28S_reference sequence

```
                                      10        20        30        40        50        60        70        80        90       100       110       120       130       140       150
ZT_5.8S-ITS2-28S_Reference    TGGGTATTGGCGTCTTTTCGCGGGGGATCACTCCCCGCGCGCCTCAAAGTCTCCGGCTGAGCGGTCTCGTCTCCCAGCGTTGTGGCATCACGTCTCGCCGCGGAGTTCACGAGCCCTCACGGCCGTTAAATCACACCTCAGGTTGACC
ZT_HiScribe_cr:tracrRNA_Run_2 ......................N...............................................................................................................................
ZT_HiScribe_cr:tracrRNA_Run_1 ......................N...............................................................................................................................

                                     160       170       180       190       200       210       220       230       240       250       260       270       280       290       300
ZT_5.8S-ITS2-28S_Reference    TCGGATCGGGTAGGGATACCCGCTGAACTTAAGCATATCAATAAGCGGAGGAAAAGAAACCAACAGGGATTGCCCTAGTAACGGCGAGTGAAGCGGCAACAGCTCAAATTTGAAATCTGGCCCCCCGGCCCGAGTTGTAATTTGTAGAGG
ZT_HiScribe_cr:tracrRNA_Run_2 ........................................................................................................................N.............................
ZT_HiScribe_cr:tracrRNA_Run_1 ........................................................................................................................N.............................

                                     310       320       330       340       350       360       370       380       390       400       410       420       430       440       450
ZT_5.8S-ITS2-28S_Reference    ATGCTTCTGGGTAGCGACCGGTCTAAGTTCCTTGGAACAGGACGTCATAGAGGGTGAGAATCCCGTATGCGACCGGCCCGCGCCCTCCACGTAGCTCCTTCGACGAGTCGAGTTGTTTGGGAATGCAGCTCTAAATGGGAGGTAAATTTC
ZT_HiScribe_cr:tracrRNA_Run_2 ......................................................................................................................................................
ZT_HiScribe_cr:tracrRNA_Run_1 ......................................................................................................................................................

                                     460       470       480       490       500       510       520       530       540       550       560       570       580       590       600
ZT_5.8S-ITS2-28S_Reference    TTCTAAAGCTAAATACCGGCCAGAGACCGATAGCGCACAAGTAGAGTGATCGAAAGATGAAAAGCACTTTGGAAAGAGAGTTAAAAAGCACGTGAAATTGTTGAAAGGGAAGCGCTTACAACCAGACTTTGGGGCGGTGTTCCGCCGGTC
ZT_HiScribe_cr:tracrRNA_Run_2 ......................................................................................................................................................
ZT_HiScribe_cr:tracrRNA_Run_1 ......................................................................................................................................................

                                     610       620       630       640       650       660       670       680       690       700       710       720       730       740       750
ZT_5.8S-ITS2-28S_Reference    TTCTGACCGGTCTACTCTCCGTCCCGAGGCCAACATCATCTGGGACCGCCGGACAAGACCTCAGGAATGTAGCTCCCCCTCGGGGGAGTGTTATAGCCTGTGGTGATGCGGCGCGTCCCGGGTGAGGTCCGCGCTTCGGCAAGGATGTTG
ZT_HiScribe_cr:tracrRNA_Run_2 ......................................................................................................................................................
ZT_HiScribe_cr:tracrRNA_Run_1 ......................................................................................................................................................

                                     760       770       780       790       800       810       820       830       840       850       860       870       880       890       900
ZT_5.8S-ITS2-28S_Reference    GCGTAATGGTTGTCAGCGGCCCGTCTTGAAACACGGACCAAGGAGTCTAACATCTATGCGAGTGTTCGGGTGTCAAACCCCTACGCGGAATGAAAGTGAACGGAGGTGGGAAGGGGCAACCCTGCACCATCGACCGATCCTGATGTCCTC
ZT_HiScribe_cr:tracrRNA_Run_2 ......................................................................................................................................................
ZT_HiScribe_cr:tracrRNA_Run_1 ......................................................................................................................................................

                                     910       920       930       940       950       960       970       980       990      1000      1010      1020      1030      1040      1050
ZT_5.8S-ITS2-28S_Reference    GGATGGATTTGAGTAAGAGCATAGCTGTTGGGACCCGAAAGATGGTGAACTATGCCTGAATAGGGTGAAGCCAGAGGAAACTCTGGTGGAGGCTCGCAGCGGTTCTGACGTGCAAATCGATCGTCAAATTTGGGTATAGGGGCGAAAGAC
ZT_HiScribe_cr:tracrRNA_Run_2 ......................................................................................................................................................
ZT_HiScribe_cr:tracrRNA_Run_1 ......................................................................................................................................................

                                    1060      1070      1080      1090      1100      1110      1120      1130      1140      1150      1160      1170      1180      1190      1200
ZT_5.8S-ITS2-28S_Reference    TAATCGAACCATCTAGTAGCTGGTTCCTGCCGAAGTTTCCCTCAGGATAGCAGTAACGTTTTCAGTTTTATGAGGTAAAGCGAATGATTAGAGGCCTTGGGGTTGAAACAACCTTAACCTATTCTCAAACTTTAAATATGTAAGAAGTCC
ZT_HiScribe_cr:tracrRNA_Run_2 ......................................................................................................................................................
ZT_HiScribe_cr:tracrRNA_Run_1 ......................................................................................................................................................

                                    1210      1220      1230      1240      1250      1260      1270      1280      1290      1300      1310      1320      1330      1340      1350
ZT_5.8S-ITS2-28S_Reference    TTGTTACTTAGTTGAACGTGGACATTTGAATGTATCGTTACTAGTGGGCCATTTTTGGTAAGCAGAACTGGCGATGCGGGATGAACCGAACGCGAGGTTAAGGTGCCGGAATGTACGCTCATCAGACACCACAAAAGGTGTTAGTTCATC
ZT_HiScribe_cr:tracrRNA_Run_2 ......................................................................................................................................................
ZT_HiScribe_cr:tracrRNA_Run_1 ......................................................................................................................................................

                                    1360      1370      1380      1390      1400      1410      1420      1430      1440      1450      1460      1470      1480      1490      1500
ZT_5.8S-ITS2-28S_Reference    TAGACAGCAGGACGGTGGCCATGGAAGTCGGAATCCGCTAAGGAGTGTGTAACAACTCACCTGCCGAATGAACTAGCCCTGAAAATGGATGGCGCTTAAGCGTGCTACCCATACCTCGCCGCCAGGGTAGAAACGATGCCCTGGCGAGTA
ZT_HiScribe_cr:tracrRNA_Run_2 ......................................................................................................................................................
ZT_HiScribe_cr:tracrRNA_Run_1 ......................................................................................................................................................

                                    1510      1520      1530      1540      1550
ZT_5.8S-ITS2-28S_Reference    GGCAGGCGTGGAGGTCCGTGACGAAGCCTTGGGGGTGACCCCGGGTCGAACGGCCTCTA
ZT_HiScribe_cr:tracrRNA_Run_2 ..............................N............................
ZT_HiScribe_cr:tracrRNA_Run_1 ..............................N............................
```

S1 Table 8. Alignment of PGT_HiScribe_cr:tracrRNA consensus sequences from run 1 and 2 to the PST_5.8S-ITS2-28S_reference sequence

```
                                        10        20        30        40        50        60        70        80        90       100       110       120       130       140       150
PGT_5.8S-ITS2-28S_Reference    TGAGTGTCATGAAACCCTCTCATTAAACATTTTTTTAATAATTTGTTTATTGGATGTTGAGTGTTGCTATAATTATAGCTCACTTTAAATATATAAGTCACTTTTCAAATAAGTTGGATTGACTTGGTGTAATAATTTTATCATCACATC
PGT_HiScribe_cr:tracrRNA_Run_2 .............................N........................................................................................................................
PGT_HiScribe_cr:tracrRNA_Run_1 .............................N........................................................................................................................

                                       160       170       180       190       200       210       220       230       240       250       260       270       280       290       300
PGT_5.8S-ITS2-28S_Reference    AAGGAAAGTAGTCAAACACTTGCCATTTTTTTTGTAACAAGAGACTCCTAAAAACCCAATATCTATTTTTTTTAAGACCTCAAATCAGGTGGGACTACCCGCTGAACTTAAGCATATCAATAAGCGGAGGAAAAGAAACTAACAAGGATTC
PGT_HiScribe_cr:tracrRNA_Run_2 .........................N.......................................N...................................................................................N
PGT_HiScribe_cr:tracrRNA_Run_1 .........................N.......................................N...................................................................................N

                                       310       320       330       340       350       360       370       380       390       400       410       420       430       440       450
PGT_5.8S-ITS2-28S_Reference    CCCTAGTAACGGCGAGTGAAGAGGGAAAAGCCCAAATTTGTAATCTGGCTCTTTCAGAGTCCGAGTTGTAATTTTAAGAACTGTTTTCAGTGCTGGACCATGTATAAGTCTGTTGAAAAGCAGCATCATTGAGGGTGATAATCCCGTTTA
PGT_HiScribe_cr:tracrRNA_Run_2 ......................................................................................................................................................
PGT_HiScribe_cr:tracrRNA_Run_1 ......................................................................................................................................................

                                       460       470       480       490       500       510       520       530       540       550       560       570       580       590       600
PGT_5.8S-ITS2-28S_Reference    TGATATGGACTACCAGTGCATTATGATACAGTCTCTAAGAGTCGAGTTGTTTGGGAATGCAGCTCAAAGTGGGTGGTAAATTCCATCTAAGGCTAAATATAGGTGAGAGACCGATAGCAAACAAGTACCGTGAGGGAAAGATGAAAAGAA
PGT_HiScribe_cr:tracrRNA_Run_2 ......................................................................................................................................................
PGT_HiScribe_cr:tracrRNA_Run_1 ......................................................................................................................................................

                                       610       620       630       640       650       660       670       680       690       700       710       720       730       740       750
PGT_5.8S-ITS2-28S_Reference    CTTTGGAAAGAGAGTTAACAGTACGTGAAATTGTTAAAAGGGAAACACTTGAAGTTAGACTTGTTATTATTAGTTCAACCTTTTTATTAAGGGAGTATTCTAATGATTAACAGACCAACATCAATTTTTGAGTGTTGGAGAAGGGTTTAA
PGT_HiScribe_cr:tracrRNA_Run_2 ......................................................................................................................................................
PGT_HiScribe_cr:tracrRNA_Run_1 ......................................................................................................................................................

                                       760       770       780       790       800       810       820       830       840       850       860       870       880       890       900
PGT_5.8S-ITS2-28S_Reference    GGAAATGTAGCAGTCTTTGGCTGTGTTATAGTCCTGAGCTTTGATACAATGCTTAAGATTGAGGAAGGCAGTAAGCGCAATTTATTGTGTGGAACAAATTAATGTTCTTCTTACTGAGGATGTTGGTGTAATAGCTTTAAATGACCCGTC
PGT_HiScribe_cr:tracrRNA_Run_2 ......................................................................................................................................................
PGT_HiScribe_cr:tracrRNA_Run_1 ......................................................................................................................................................

                                       910       920       930       940       950       960       970       980       990      1000      1010      1020      1030      1040      1050
PGT_5.8S-ITS2-28S_Reference    TTGAAACACGGACCAAGGAGTCTAACATGCTTGCAAGTATTTGGGTGCTTGAAACCCTTATGCGTAATGAAAGTAAATGTAAATGGGATCTGTCAAAAGTGCACCATTGACCAGTCCAGATTATTTATATGATGGTACTGAGTAAGAGCA
PGT_HiScribe_cr:tracrRNA_Run_2 ......................................................................................................................................................
PGT_HiScribe_cr:tracrRNA_Run_1 ......................................................................................................................................................

                                      1060      1070      1080      1090      1100      1110      1120      1130      1140      1150      1160      1170      1180      1190      1200
PGT_5.8S-ITS2-28S_Reference    AGTATGTTGGGACCCGAAAGATGGTGAACTATGCCTGAATAGGGGTGAAGCCAGAGGAAACTCTGGTGGAAGCTCGTAGCGGTTCTGACGTGCAAATCGATCGTCAAATTTGGGTATAGGGGCGAAAGACTAATCGAACCATCTAGTAGC
PGT_HiScribe_cr:tracrRNA_Run_2 ......................................................................................................................................................
PGT_HiScribe_cr:tracrRNA_Run_1 ......................................................................................................................................................

                                      1210      1220      1230      1240      1250      1260      1270      1280      1290      1300      1310      1320      1330      1340      1350
PGT_5.8S-ITS2-28S_Reference    TGGTTCCTGCCGAAGTTTCCCTCAGGATAGCAAAGACTCGTATCAGTTTTATGAGGTAAAGCGAATGATTAGAGGCCTTGGGGATGAAACATCCTTAACCTATTCTCAAACTTTAAATATGTAAGACGCTCCTGTTTCTTAATTGAACGT
PGT_HiScribe_cr:tracrRNA_Run_2 ......................................................................................................................................................
PGT_HiScribe_cr:tracrRNA_Run_1 ......................................................................................................................................................

                                      1360      1370      1380      1390      1400      1410      1420      1430      1440      1450      1460      1470      1480      1490      1500
PGT_5.8S-ITS2-28S_Reference    GGGCATGTGAATGAGAGTCTTTAGTGGGCCATTTTTGGTAAGCAGAACTGGCGATGCGGGATGAACCGAACGTGAGGTTAAGGTGCCGGAATATACACTCATCAGACACTACAAAAGGTGTTAGTTCATCTAGACAGCCGCACGGTGGCC
PGT_HiScribe_cr:tracrRNA_Run_2 ......................................................................................................................................................
PGT_HiScribe_cr:tracrRNA_Run_1 ......................................................................................................................................................

                                      1510      1520      1530      1540      1550      1560      1570      1580      1590      1600      1610      1620      1630      1640      1650
PGT_5.8S-ITS2-28S_Reference    ATGGAAGTCGGAATCCGCTAAGGAGTGTGTAACAACTCAACGGCCGAATGAACTAGCCCTGAAAATGGATGGCGCTTAAGTGTATTACCCATACCTCACCATTAATATTGTTTTTCATTACATATTAATGAGTAGGCAGGCGTGGAGGTT
PGT_HiScribe_cr:tracrRNA_Run_2 ......................................................................................................................................................
PGT_HiScribe_cr:tracrRNA_Run_1 ......................................................................................................................................................

                                      1660      1670      1680      1690
PGT_5.8S-ITS2-28S_Reference    ATGTAGCGAAGCCTTGGCAGTGATGCTGGGTGGAACAGCCTCTA
PGT_HiScribe_cr:tracrRNA_Run_2 ............................................
PGT_HiScribe_cr:tracrRNA_Run_1 ............................................
```

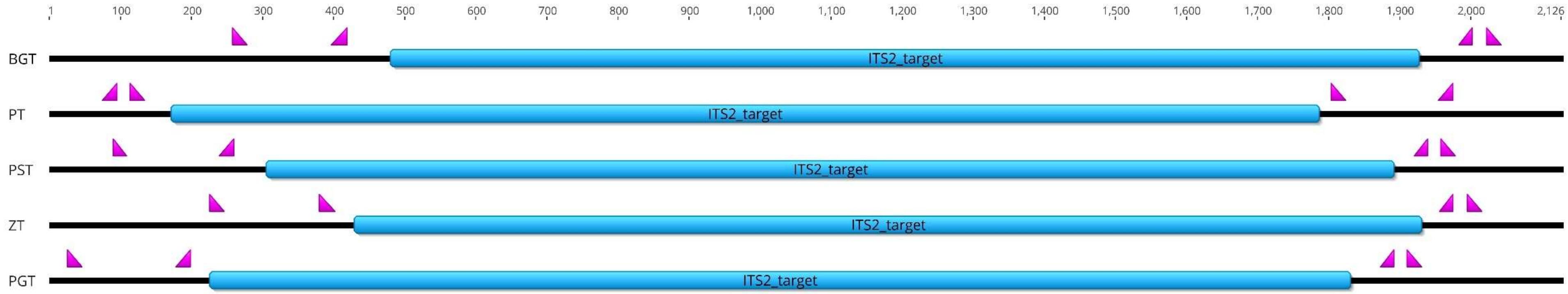

S1 Fig 4. Schematic diagram of Cas9 gRNA directionalities on the five ITS2 pathogen target sequences. The ITS2 target region is shown in blue. Each gRNA is depicted as a pink triangle, with the pointy end of the triangle positioned closest to the PAM sequence. The target sequences are labelled as BGT, PT, PST, ZT, and PGT.
